# MDGraphEmb: a toolkit for graph embedding and classification of protein conformational ensembles

**DOI:** 10.1093/bioinformatics/btaf420

**Published:** 2025-07-31

**Authors:** Ferdoos Hossein Nezhad, Namir Oues, Massimiliano Meli, Alessandro Pandini

**Affiliations:** Department of Computer Science, Brunel University of London, Uxbridge UB8 3PH, United Kingdom; Department of Computer Science, Brunel University of London, Uxbridge UB8 3PH, United Kingdom; Istituto di Scienze e Tecnologie Chimiche “Giulio Natta”—SCITEC, Consiglio Nazionale delle Ricerche, Milano 20131, Italy; Department of Computer Science, Brunel University of London, Uxbridge UB8 3PH, United Kingdom; The Thomas Young Centre for Theory and Simulation of Materials, London SW7 2AZ, United Kingdom

## Abstract

**Motivation:**

Molecular Dynamics (MD) simulations are essential for investigating protein dynamics and function. Although significant advances have been made in integrating simulation techniques and machine learning, there are still challenges in selecting the most suitable data representation for learning. Graph embedding is a powerful computational method that automatically learns low-dimensional representations of nodes in a graph while preserving graph topology and node properties, thereby bridging graph structures and machine learning methods. Graph embeddings hold great potential for efficiently representing MD simulation data and studying protein dynamics.

**Results:**

We present MDGraphEmb, a Python library built on MDAnalysis, specifically designed to convert protein MD simulation trajectories into graph-based representations and corresponding graph embeddings. This transformation enables the compression of high-dimensional, noisy trajectories from protein simulations into tabular formats suitable for machine learning. MDGraphEmb provides a framework that supports a range of graph embedding techniques and machine learning models, enabling the creation of workflows to analyse protein dynamics and identify important protein conformations. Graph embedding effectively captures and compresses structural information from protein MD simulation data, making it applicable to diverse downstream machine-learning classification tasks. We present an application for encoding and detecting important protein conformations from molecular dynamics simulations to classify functional states, using adenylate kinase (ADK) as the main case study. To assess the generalizability of the approach, two additional systems, Plantaricin E (PlnE) and HIV-1 protease are included as supplementary validation examples. A performance comparison of different graph embedding methods combined with machine learning models is also provided.

**Availability and implementation:**

MDGraphEMB GitHub Repository: https://github.com/FerdoosHN/MDGraphEMB.

## 1 Introduction

Proteins are at the core of most biological processes. Their ability to adopt different conformations is essential for several biological processes, including enzymatic activity, signalling, genetic information processing, transport and trafficking, immune response, and cellular homeostasis mechanisms. Investigating these processes often requires identifying important protein conformations associated with functional states and describing the mechanisms of transition between these states.

In this regard, MD simulations have become a routine tool for studying protein dynamics and exploring their conformational space (Henzler-Wildman and Kern 2007). These simulations provide information on events at the atomic and molecular levels, capturing processes up to the millisecond scale. Moreover, MD simulation techniques are frequently used in combination with experimental methods to investigate functional dynamics in proteins. Similar to other areas of data-driven research, the availability of faster algorithms and high-performance parallelization has made it easier to study more complex problems over extended timescales, often approaching those observed in experimental techniques. Recently, machine learning techniques have demonstrated great potential for analysing and extracting insights from the extensive data generated by MD simulations (Glielmo *et al.* 2021, Kaptan and Vattulainen 2022, Hagg and Kirschner 2023). However, molecular simulation data, particularly in the case of atomistic molecular dynamics, has a low signal-to-noise ratio, making the detection and modelling of functionally relevant motions challenging. As a result, dimensionality reduction and data compression are increasingly used for extracting biologically meaningful patterns and enabling efficient, quantitative interpretation through machine learning. Historically, unsupervised machine learning methods have been used to extract meaningful information from conformational landscapes, particularly through dimensionality reduction (Glielmo *et al.* 2021). Approaches based on machine and deep learning have been developed to handle dimensionality reduction and compress information from MD simulation data, facilitating effective learning (Lemke and Peter 2019, Lemke *et al.* 2019). Recently, an autoencoder (Jin *et al.* 2021) was used to map MD simulation snapshots to a conformational landscape defined by principal component analysis, enabling the prediction of new conformations not included in the training data.

While dimensionality reduction methods can enhance feature extraction, they require a thorough analysis of the conformational space. Alternative approaches focus on modifying the data representation of each conformation to generate more informative representations of key degrees of freedom and their relationships. In this context, the spatial arrangement and interactions of protein residues have been effectively represented using graph models (Patel *et al.* 2024). These models have been particularly successful in capturing the multiscale nature of intrinsic dynamics and conformational motions, accurately modelling both global and local changes, as well as complex time-resolved events such as allosteric communication.

Graph representations have been used successfully to encode and analyse the conformational properties of protein structures (Patel *et al.* 2024). For residue-based representations, a single graph was typically constructed from average information on residue-residue interactions or dynamical coupling, given an ensemble of conformations. An alternative approach involves encoding the ensemble as a collection of graphs. However, the heterogeneity and dynamic nature of conformational ensembles, combined with the complexity of high-dimensional data, present significant challenges for traditional graph analysis.

Graph embedding techniques address these challenges by converting graph data into low-dimensional vector representations while preserving key structural information, enabling more efficient and effective analysis. In addition, graph embedding techniques offer significant computational advantages over methods that operate directly on the original networks. They facilitate faster processing and can be used to build machine learning models for various predictive tasks, such as node classification, community detection, clustering, and visualization (Hamilton *et al.* 2017, Yue *et al.* 2020). Additionally, working in a lower-dimensional space helps to manage noise present in the original network more effectively. Modelling using the graph embedding developed in recent years (Grover and Leskovec 2016, Hamilton *et al.* 2017, Zhang *et al.* 2018, Cui *et al.* 2019, Yue *et al.* 2020). In general, graph embedding methods can be categorized into three main types: spectral-based, random walk-based, and neural network-based methods (Nelson *et al.* 2019, Yue *et al.* 2020). The field of graph learning has expanded significantly, progressing beyond traditional graph representation learning and neural network-based embedding techniques. This growth has driven the development and evaluation of various Graph Neural Network (GNN) architectures, including Graph Convolutional Network (GCN) (Kipf and Welling 2017), Graph Sample and Aggregate (GraphSAGE) (Hamilton *et al.* 2017), and Graph Attention Network (GAT) (Veličković *et al.* 2020), each designed to address specific challenges in graph-based tasks. These models have been successfully applied to a wide range of applications (Zitnik and Leskovec 2017, Jin *et al.* 2018, You *et al.* 2018). Moreover, platforms such as GraphGym and PyTorch Geometric (PyG) (Fey and Lenssen 2019, You *et al.* 2020) have emerged as powerful tools for exploring and benchmarking various GNN architectures and tasks. Deep Graph Library (DGL) (Wang *et al.* 2020b) complements these efforts with a graph-centric design that supports efficient parallel computation through generalized sparse tensor operations. A comprehensive survey on graph embedding techniques and graph representation learning in bioinformatics, detailing trends, methods, and applications, can be found in Wu *et al.* (2023) and Yi *et al.* (2022). In recent years, graph embedding and graph learning techniques have been increasingly applied to predict protein function by leveraging information from protein sequences, structures, and interaction networks (Lin *et al.* 2024, Boadu *et al.* 2025). However, these methods have yet to be thoroughly tested or analysed in the context of protein dynamics and MD simulation data.

In this paper, we explore graph embedding as an effective computational method for learning low-dimensional node representations of ensembles of protein structure graphs from molecular dynamics. To this end, we developed MDGraphEmb, an object-oriented Python library built on top of the MDAnalysis library (Michaud-Agrawal *et al.* 2011, Gowers *et al.* 2019). MDGraphEmb facilitates the conversion of MD simulation data from protein conformations into graph representations and then into graph embeddings using different embedding methods. The tool also supports a range of machine learning and deep learning models to create predictors of important protein conformations from MD simulation data. We demonstrate that graph embedding effectively captures structural information from MD simulation data, making it suitable for different machine learning classification tasks. An example application using the ADK protein system is presented for encoding and detecting important protein conformations from molecular dynamics simulations to classify functional states. Within this context, we compare the performance of various graph embedding methods in combination with machine learning models to evaluate their effectiveness. Based on these results, we recommend the most effective embedding and classification strategies. To assess the generalizability of the approach, two additional systems (PlnE and HIV-1 protease) are included as supplementary validation examples.

Conformational states are often identified using unsupervised learning methods such as clustering analysis. While effective as an exploratory analysis tool, clustering relies on similarity-based grouping and often struggles to distinguish continuous transitions between conformational states, especially in complex, high-dimensional datasets. Moreover, it typically requires ad-hoc parameter selection, and the resulting groupings are not directly transferable to new simulation data. The supervised learning approach presented in this study offers a way to overcome these limitations and complement clustering. If representative state labels can be derived from one simulation, the trained model can then be used to predict conformational states in new, unseen datasets.

While graph learning frameworks such as PyG and DGL offer extensive support for implementing graph neural networks and embedding techniques, they are general-purpose libraries not specifically optimized for the unique challenges posed by MD simulation data. In contrast, MDGraphEmb is an open-source library specifically developed to provide a domain-specific workflow for analysing protein MD simulations through graph-based learning. It integrates widely used methods (including Node2Vec, GCN, GAT, and GraphSAGE) within a unified framework that operates directly on MDAnalysis-compatible trajectory data. MDGraphEmb streamlines the entire process, from trajectory preprocessing and graph construction to representation learning and downstream classification, enabling domain experts in computational biology to apply graph learning without requiring deep expertise in graph neural network programming. It also offers pre-configured workflows, protein-specific graph construction routines, and seamless pipelines for embedding and classification, along with built-in tools for benchmarking and comparative evaluation.

## 2 Materials and methods

### 2.1 Python library architecture

MDGraphEmb is an object-oriented Python library for analysing protein dynamics using graph embeddings. Library functions are included to: (i) convert protein conformations from MD trajectories into graph representations; (ii) generate graph embeddings using well-established embedding methods; and (iii) train machine and deep learning models to predict functional properties of protein conformations. The library was built on top of MDAnalysis and incorporates functions from NetworkX (Hagberg *et al.* 2008), PyTorch Geometric (Fey and Lenssen 2019), TensorFlow (Abadi *et al.* 2016), and scikit-learn (Pedregosa *et al.* 2011). At its core, MDGraphEmb provides a flexible set of classes for handling protein conformations using three different representations: **Protein**, **ProteinGraph**, and **ProteinEmb**.

**Figure 1.**
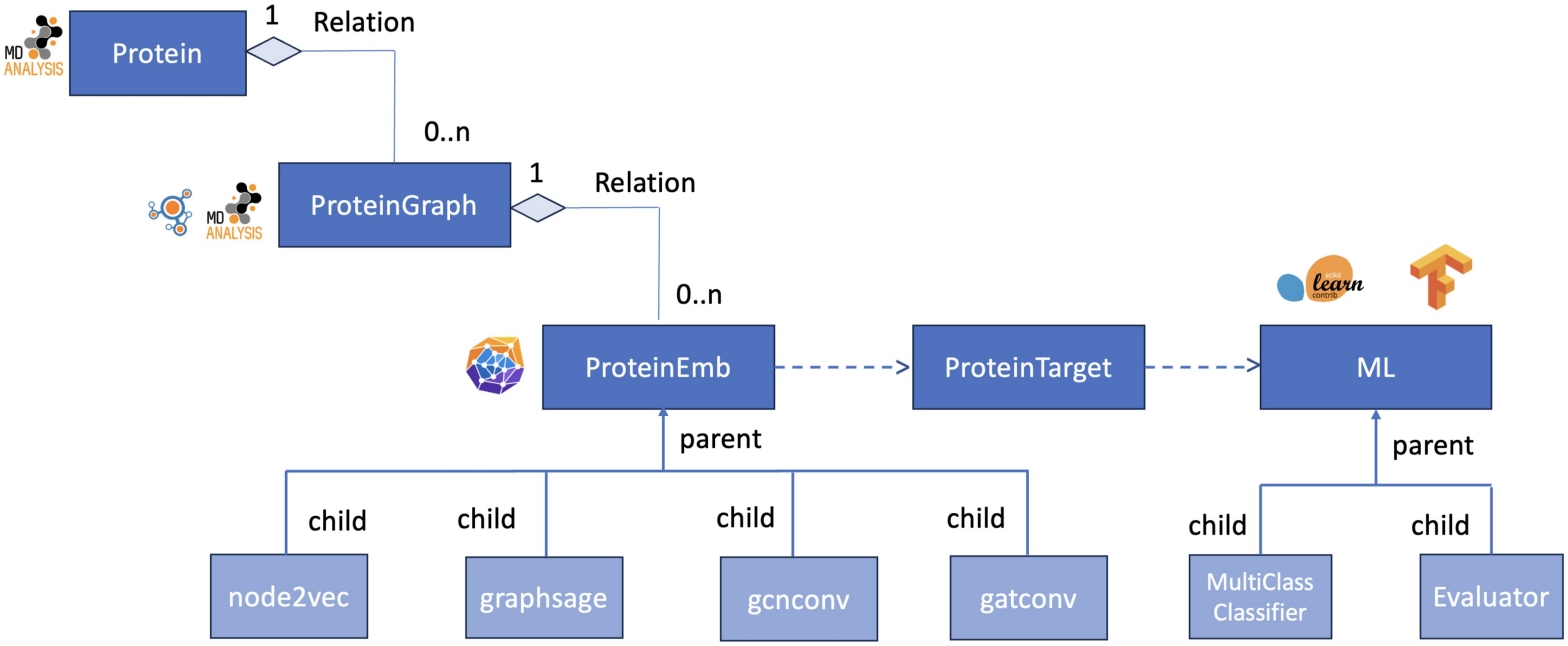
Class diagram of the MDGraphEmb toolkit. A detailed explanation can be found in the Section 2.

The **Protein** class uses the MDAnalysis Universe to represent Cartesian and topological information on protein conformations extracted from MD trajectory files, following a previous class design (Oues *et al.* 2023). The **ProteinGraph** class converts these protein conformations into graphs, preserving the spatial relationships between atoms in protein data. In more detail, for each frame, the self-distance array from MDAnalysis is converted into a weighted adjacency matrix, and a graph is generated using NetworkX. A cut-off is applied to the adjacency matrix to filter contact connections. Through this process, the MD trajectory is converted into a series of graphs. The **ProteinEmb** class compresses each graph into an embedding. It supports different graph embedding methods to transform high-dimensional graph data into lower-dimensional embeddings. This process not only reduces computational complexity but also allows for the extraction of meaningful features that are critical for machine learning tasks. To this end, the library also includes a **ProteinTarget** class, which can record per-frame (i.e. per-graph) target properties to predict. A **ML** class is provided for convenience, which offers direct access to various supervised learning algorithms. These algorithms, alongside evaluation methods, visualization tools, and report generation capabilities, facilitate the development of a workflow for training and prediction of conformational properties, e.g. functional state labels, based on learned graph embeddings. The class diagram of MDGraphEmb is presented in Fig. 1.

### 2.2 Graph embedding and machine learning prediction

**Figure 2.**
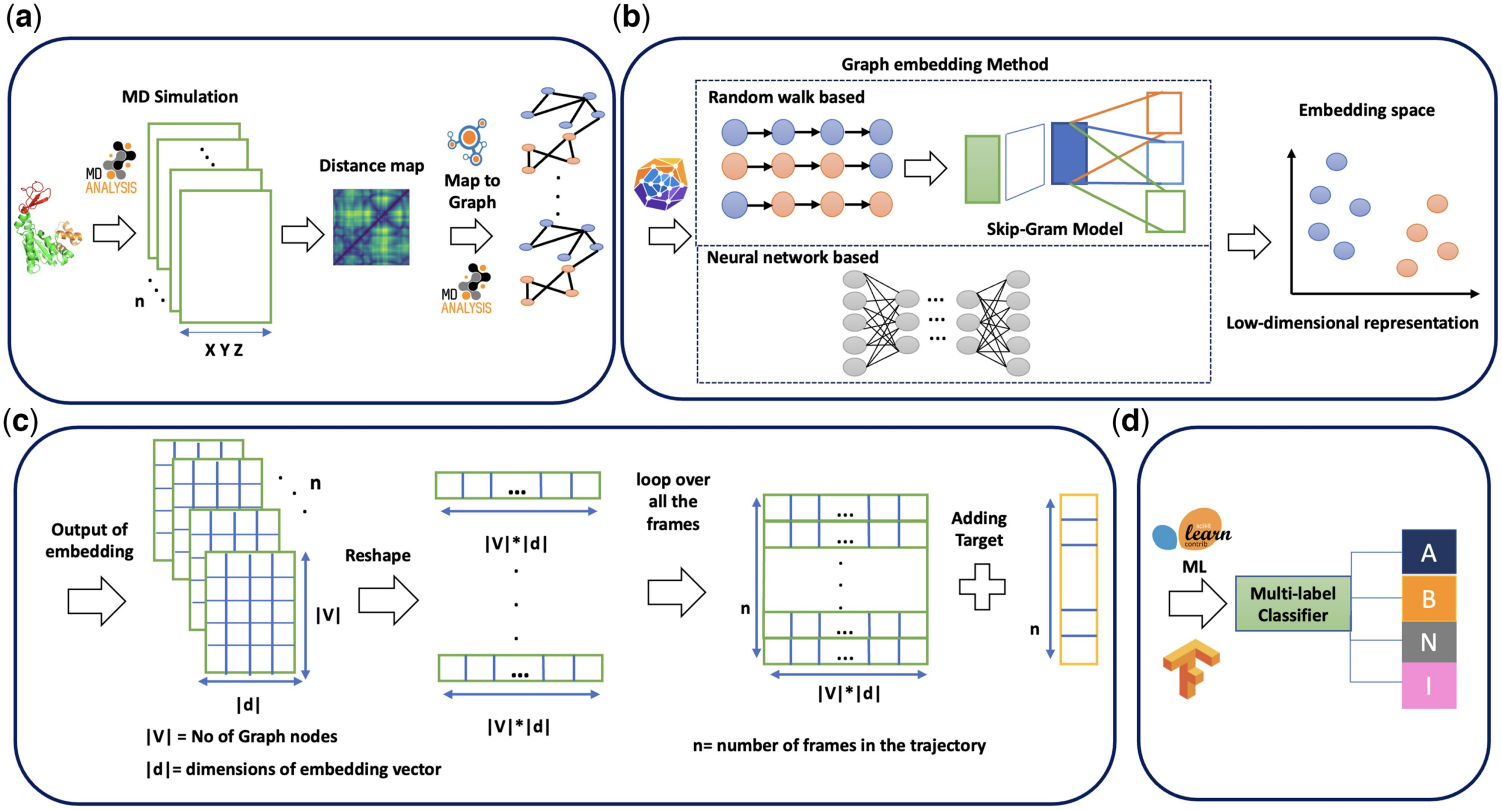
Workflow of the MDGraphEmb toolkit. (a) Protein simulation data is read by MDAnalysis, where (X,Y,Z) are Cα atom coordinates and *n* is the number of frames in protein trajectory. The protein data is converted into graph using the Cα distance matrix, with the adjacency matrix constructed using a default cutoff of 10 Å. Graphs for each frame are then generated using the NetworkX library. (b) The graphs are embedded using PyTorch Geometric, with options for various embedding methods. (C) The embedding produces a series of matrices, each of size |V|×|d|, where |V| is the number of nodes and |d| is the embedding dimension. Each frame in the trajectory results in one matrix, which is reshaped into a vector. The trajectory is represented in a tabular data format where a per-frame target value can be added. (d) This data structure is ready for machine learning classification tasks. In this paper, a classification of protein functional states is presented.

The workflow of the MDGraphEmb toolkit is shown in Fig. 2. Protein simulation data is read using MDAnalysis (Fig. 2a), where the coordinates of the Cα atoms in the protein are represented by an (X,Y,Z) matrix, and *n* denotes the number of frames in the protein trajectory. For each frame, a pairwise Cα distance matrix is calculated, filtered by a contact cut-off (default: values <10Å) (Gligorijević *et al.* 2021), and converted into a weighted graph, where edge weights are calculated as (1−(distance/cut-off)).

The resulting graphs are then embedded using the PyTorch Geometric package (Fig. 2b). Users can select from different embedding methods or configure their own hyperparameters. Both random walk-based methods (e.g. node2vec) and neural network-based methods (e.g. GraphSAGE, GAT, GCN) are supported. The embedding generates a lower-dimensional representation of the protein’s trajectory space (Fig. 2c). The embedding output is a series of matrices with dimensions |V|×|d|, where |V| represents the number of nodes in the graph (equivalent to the number of Cα) and |d| represents the dimension of the embedded vectors for each frame. Since there are *n* frames in the trajectory, we obtain *n* matrices of size |V|×|d|. The matrix output from the embedding model is reshaped for each frame into a single vector of size |V|×|d|, effectively mapping the entire graph for one frame to a single vector. The trajectory data is compressed into a tabular format conveniently processed by most machine learning algorithms. If an example of a per-frame property of interest is available, a predictive model can be trained using supervised learning methods. Parameter settings for the graph embedding methods and configurations of the machine learning models are provided in the Supplementary Materials (Methods: Architectures of the Embedding Methods and Machine Learning Models Supplementary Materials), available as supplementary data at *Bioinformatics* online.

This toolkit supports a variety of supervised machine learning algorithms through scikit-learn and TensorFlow (Fig. 2d), including neural networks (NN), (CNN), and boosting methods like LightGBM (LGBM) and XGBoost (XGB), which are well suited to high-dimensional data. Additionally, it accommodates traditional machine learning techniques such as logistic regression, random forests, and support vector machines. The toolkit provides a comprehensive graph learning framework encompassing the entire process, from embedded protein data to target processing, classification reporting, visualization, and performance comparison between different embedding methods and machine learning models.

### 2.3 Case study: system description

**Figure 3.**
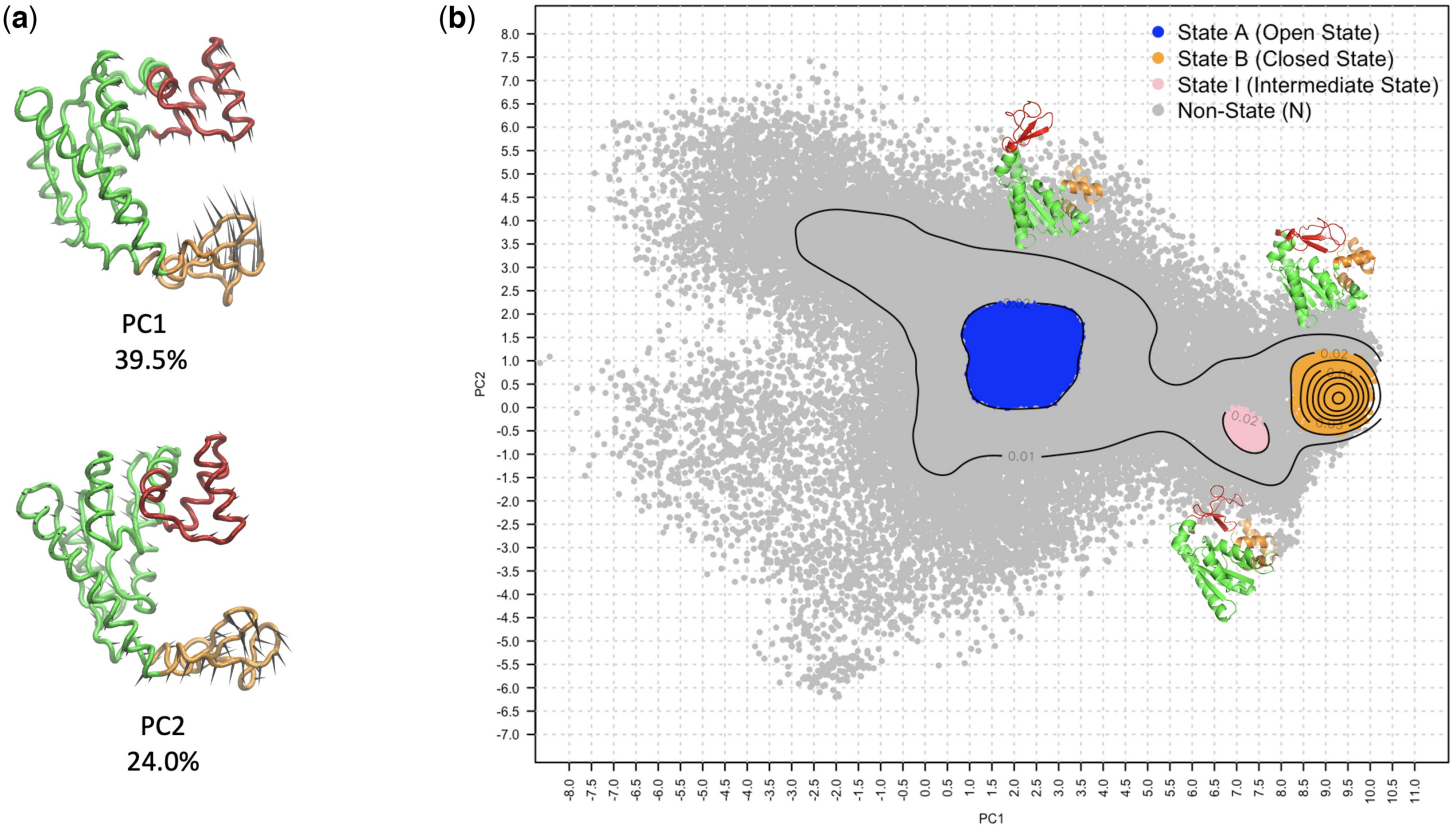
Machine learning target labels for the ADK conformational classification scenario, derived using PCA on MD trajectory data. (a) PC1 (see also *x*-axis in panel b) describes open–closed conformational transition in ADK, while PC2 (*y*-axis) describes the twisting motion of the LID domain. The structural architecture of ADK comprises three domains: the CORE domain (green; residues 1–29, 68–117, 161–214), the NMPbind domain (red; residues 30–67), and the LID domain (orange; residues 118–160). While the CORE domain acts as a stable scaffold, the NMPbind and LID domains undergo conformational changes, closing over ligand binding sites during catalysis. (b) A density contour plot estimates the distribution of conformational states in this PCA space. Based on density thresholds, four hypothetical states were defined: open (A), closed (B), intermediate (I), and non-state (N).

Adenylate kinase (ADK) is an enzyme within the phosphotransferase family, playing a central role in maintaining cellular energy balance by catalysing the conversion of adenine nucleotides. A critical step in the catalytic cycle is the transition between an open and a closed state. This conformational transition makes ADK an ideal model system for studying protein conformational changes due to its well-defined states. Details of the open-close conformational transition have been extensively investigated through combinations of experimental and computational methods (Henzler-Wildman *et al.* 2007, Daily *et al.* 2010, Ping *et al.* 2013, Formoso *et al.* 2015, Wang *et al.* 2020a). ADK dynamics is compatible with an induced-fit model and a complete transition to a closed state is generally observed in the presence of the substrate. A recent computational study demonstrated that a double mutant (V135G, V142G) shows features of pre-existing equilibrium and can sample the closed state in the absence of the substrate (Song *et al.* 2021). Mutations at residues V135 and V142 are located on the lid domain of ADK, in a flexible loop region directly involved in the conformational transition. The structural architecture of ADK is illustrated in Fig. 3.

In addition to the ADK case study, MDGraphEmb was further evaluated on two structurally and dynamically distinct systems—PlnE and HIV-1 protease, to assess the generalizability of the approach. PlnE is an α-helical antimicrobial peptide with high conformational flexibility, while HIV-1 protease exhibits pronounced flap dynamics that regulate access to its active site. These systems were selected to test the method across different types of conformational dynamics. Full details are provided in the Supplementary Materials (Case Study Systems: Preparation and Simulation), available as supplementary data at *Bioinformatics* online.

### 2.4 System preparation and simulation

The wild-type structure of ADK was downloaded from the Protein Data Bank (Berman *et al.* 2003). A double mutant (V135G + V142G) was generated from this structure (PDB ID: 4AKE) using PyRosetta (Chaudhury *et al.* 2010). Sampling of conformational changes in ADK was done by MD simulation using GROMACS 2022.4 with the AMBER ff99SB*-ILDN force field. Details of system minimization and equilibration are reported in the Supplementary Materials (Case Study Systems: Preparation and Simulation Supplementary Materials), available as supplementary data at *Bioinformatics* online. For the wild-type and double mutant structures, 5 replicas of 1 microsecond (μs) were generated using a 2-femtosecond timestep. A dataset of 100 000 frames recorded every 10 picoseconds was created for this study from the replica R02 of the double mutant, that is the one with the most extensive transition towards the closed state, so the one more informative for effective training.

### 2.5 Conformational state prediction

The aim of this study is to demonstrate that graph embedding effectively captures information on protein conformations from MD simulation data and can be used to train machine learning models to predict conformational states. To this end, we designed a prediction scenario where a supervised learning model is trained to classify conformational states. The scenario was tested on the MD trajectory of the ADK transition from the open to the closed state.

Initially, a state label was generated for each trajectory frame using a non-trivial procedure that included expert knowledge decisions: states associated with energy minima were identified as high-density regions in the conformational space describing ADK dynamics. First, critical collective motions describing ADK dynamics were extracted using Principal Component Analysis (PCA) calculated over a combined trajectory of wild-type and double mutant simulations (Amadei *et al.* 1993). The first principal component (PC1—39.5% explained variance) clearly describes the lid closure, while the second principal component (PC2—24.0%) captures the twisting motion of the lid, known to lock the conformation in the closed state (Fig. 3a). Second, the dataset of conformations was projected onto the (PC1, PC2) space, and a density analysis was performed. Contour lines were generated, and the maximal contour threshold that separates the two basins of open and closed conformations was identified, that in the case of (PC1, PC2) space corresponded to 0.02 units of density over the total space area. Finally, boundaries for each area in the (PC1, PC2) space with a density higher than the threshold were defined. All data points (frames) within each area were labelled accordingly: open (A), closed (B), intermediate (I). All remaining data points in lower-density regions were labelled non-state (N).

While this procedure does not follow a rigorous free energy reconstruction, it offers a robust framework to test the potential of machine learning in predicting state labels. These labels are generated in a non-trivial and non-linear way, avoiding a direct dependency on the coordinates of individual frames. Additionally, the state boundaries do not define any easily calculable hyperplane in the PCA space. Information on PCA will not be used in the next steps of model training to add robustness to the test. With a suitable target variable established, a model can be trained to learn the relationship between the input conformation, as represented by the graph embedding, and the output target label representing the conformational state of that conformation (see Fig. 2c).

A set of supervised machine learning algorithms was used to train and test a classification model using different subsets of frames from the protein trajectory (5000, 10 000, 25 000, 50 000, and 100 000). Subsets were derived by striding at 200, 100, 40, 20, and 10 ps. All subsets contained frames representative of the three main conformational states. The models were trained on 70% of the data. Following training, the models were evaluated on the remaining 30% of the data to assess their predictive performance. This evaluation was conducted using different combinations of embedding techniques, machine learning models, and different dataset sizes to identify the best predictive framework.

### 2.6 Statistical analysis and visualization tools

Initial data cleaning, preparation, statistical analysis, and final plots were generated using the R statistical environment (R Core Team 2021). Density analysis and state labelling were performed using the MASS (Venables and Ripley 2002) and sp (Pebesma and Bivand 2005) libraries. Images of protein structures were generated using PyMol (Schrödinger, LLC 2015) and VMD (Humphrey *et al.* 1996).

## 3 Results and discussion

Different predictive models were trained and tested to classify conformational states at the frame level for the ADK system. Three key aspects of the data analysis workflow were investigated: (i) the choice of embedding methods, (ii) the machine learning models, and (iii) the dataset sizes (see Table 1). For details refer to the Supplementary Materials (Results section), available as supplementary data at *Bioinformatics* online. This includes a comparison of class-specific and overall accuracy across different embedding methods, as well as an evaluation of various performance metrics by class for multiple machine learning models using GraphSAGE embeddings across different frame counts. The extended analysis covers not only the ADK system, but also two additional protein systems: PlnE and HIV-1 protease. Embeddings were generated with GAT, GCN, GraphSage, and Node2Vec. Among these, GraphSage demonstrated superior performance, generating high-quality embeddings that consistently outperformed the other methods. GraphSage was ultimately selected as the optimal method due to its scalability and ability to handle high-dimensional data efficiently. Machine learning models trained on GraphSage embeddings achieved the highest overall performance, highlighting its robustness in capturing the underlying structure of the data.

**Table 1.** Comparison of class-specific and overall accuracy across different frames and machine learning models for GraphSage.

Trajectory size	ML Model	Class A	Class B	Class I	Class N	Model Accuracy
5000	Logistic Regression	**0.57**	0.95	0.43	0.74	0.74
	Random Forest	0.28	0.97	0.43	0.88	0.81
	XGBoost	0.27	0.97	**0.55**	0.90	**0.83**
	LightGBM	0.24	0.97	**0.55**	**0.92**	**0.83**
	Neural Network	0.47	**0.98**	0.48	0.81	0.78
	CNN	0.30	0.96	0.39	0.88	0.81
	Support Vector	0.31	0.97	0.30	0.89	0.82
10 000	Logistic Regression	**0.62**	0.95	0.63	0.73	0.75
	Random Forest	0.39	**0.98**	0.54	0.88	0.82
	XGBoost	0.28	**0.98**	0.58	**0.91**	0.82
	LightGBM	0.28	**0.98**	0.53	0.90	0.82
	Neural Network	0.47	0.97	**0.70**	0.85	0.81
	CNN	0.41	0.97	0.47	0.85	0.80
	Support Vector	0.34	**0.98**	0.41	**0.91**	**0.83**
25 000	Logistic Regression	**0.72**	0.96	**0.86**	0.69	0.74
	Random Forest	0.35	**0.98**	0.63	0.86	0.80
	XGBoost	0.28	0.97	0.69	**0.91**	**0.83**
	LightGBM	0.32	0.97	0.69	0.89	0.82
	Neural Network	0.58	**0.98**	0.77	0.82	0.81
	CNN	0.31	0.94	0.53	0.90	0.82
	Support Vector	0.40	0.97	0.63	0.89	**0.83**
50 000	Logistic Regression	**0.78**	**0.98**	**0.92**	0.67	0.73
	Random Forest	0.45	**0.98**	0.72	0.86	0.82
	XGBoost	0.34	**0.98**	0.78	0.90	0.84
	LightGBM	0.38	**0.98**	0.79	0.89	0.83
	Neural Network	0.52	0.97	0.72	**0.91**	**0.86**
	CNN	0.45	0.96	0.73	0.87	0.82
	Support Vector	0.50	**0.98**	0.76	0.89	0.84
100 000	Logistic Regression	**0.81**	**0.98**	**0.95**	0.67	0.74
	Random Forest	0.47	**0.98**	0.72	0.86	0.82
	XGBoost	0.40	0.96	0.68	**0.91**	0.84
	LightGBM	0.40	0.97	0.75	0.90	0.84
	Neural Network	0.68	0.97	0.71	0.87	**0.85**
	CNN	0.51	0.96	0.67	0.88	0.83
	Support Vector	0.59	0.97	0.77	0.88	**0.85**

Highest values by Class for each trajectory size are indicated in bold.

The following presents a comparative performance analysis of different machine learning models trained on embeddings generated by the GraphSage method. The evaluated models include Logistic Regression (LR), Random Forest (RF), XGB, LGBM, NN, CNN, and Support Vector Machines (SVM). Each model was assessed based on its overall performance in classification, as well as correct predictions across the single class labels: Class A (open state), Class B (closed state), Class I (intermediate state), and Class N (non-state). The evaluation was conducted across different dataset sizes (5000, 10 000, 25 000, 50 000, and 100 000 frames), providing insights into model performance with increasing data size and frequency of sampling from the original MD trajectory.

In terms of overall accuracy, NN achieved the highest accuracy for a dataset of 50 000 frames, with a performance of 0.86, indicating their robustness across all classes. LR, LGBM, and XGB also showed strong overall performance, especially at smaller and intermediate dataset sizes, making them suitable choices when prioritizing computational efficiency.

The open state (Class A) presented considerable challenges for most machine learning models to predict accurately, while LR performed exceptionally well at larger dataset sizes, achieving an accuracy of 0.78 for 50 000 frames and 0.81 for 100 000 frames. The closed state (Class B) was the easiest class to predict, with consistently high accuracy across models and trajectory sizes. Most models achieved top accuracies above 0.95 across different trajectory sizes, showcasing the well-defined and easily recognizable patterns within Class B. This aligns with what is expected: the closed state of the protein has a distinct, defined set of conformations mapping onto a well-separated region of the phase space, while the open state can appear in different geometrical arrangements, making it more challenging to identify a common pattern.

Class I was the most challenging to predict for all models compared to Classes A and B. Neural Networks achieved the highest accuracy of 0.70 at the trajectory size of 10 000, demonstrating their ability to capture the non-linear characteristics of the intermediate state. The transitional nature of Class I makes it difficult to define, as it represents an intermediate phase between the open and closed states of the protein. For larger trajectory sizes (50 000 and 100 000 frames), Logistic Regression showed improved performance with accuracies of 0.92 and 0.95, respectively. This pattern suggests that, while Class I may display complex transitional properties at smaller dataset sizes, it benefits from linear classification methods when more data is available, as LR can detect overarching trends.

**Figure 4.**
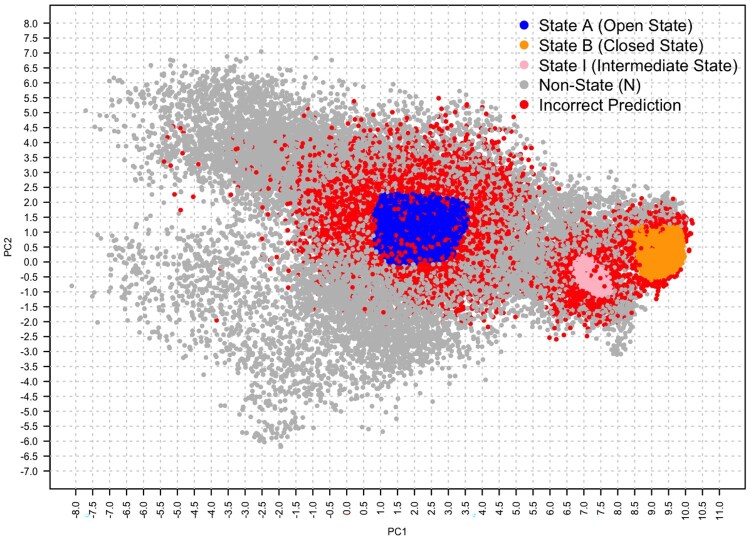
The PCA plot shows the Logistic Regression’s state predictions for 100 000 frames, using color coding to distinguish between ADK states. Red dots indicate incorrect predictions, while blue, pink, and grey dots represent correct predictions for the open (A), intermediate (I), and non-state (N), respectively.

**Figure 5.**
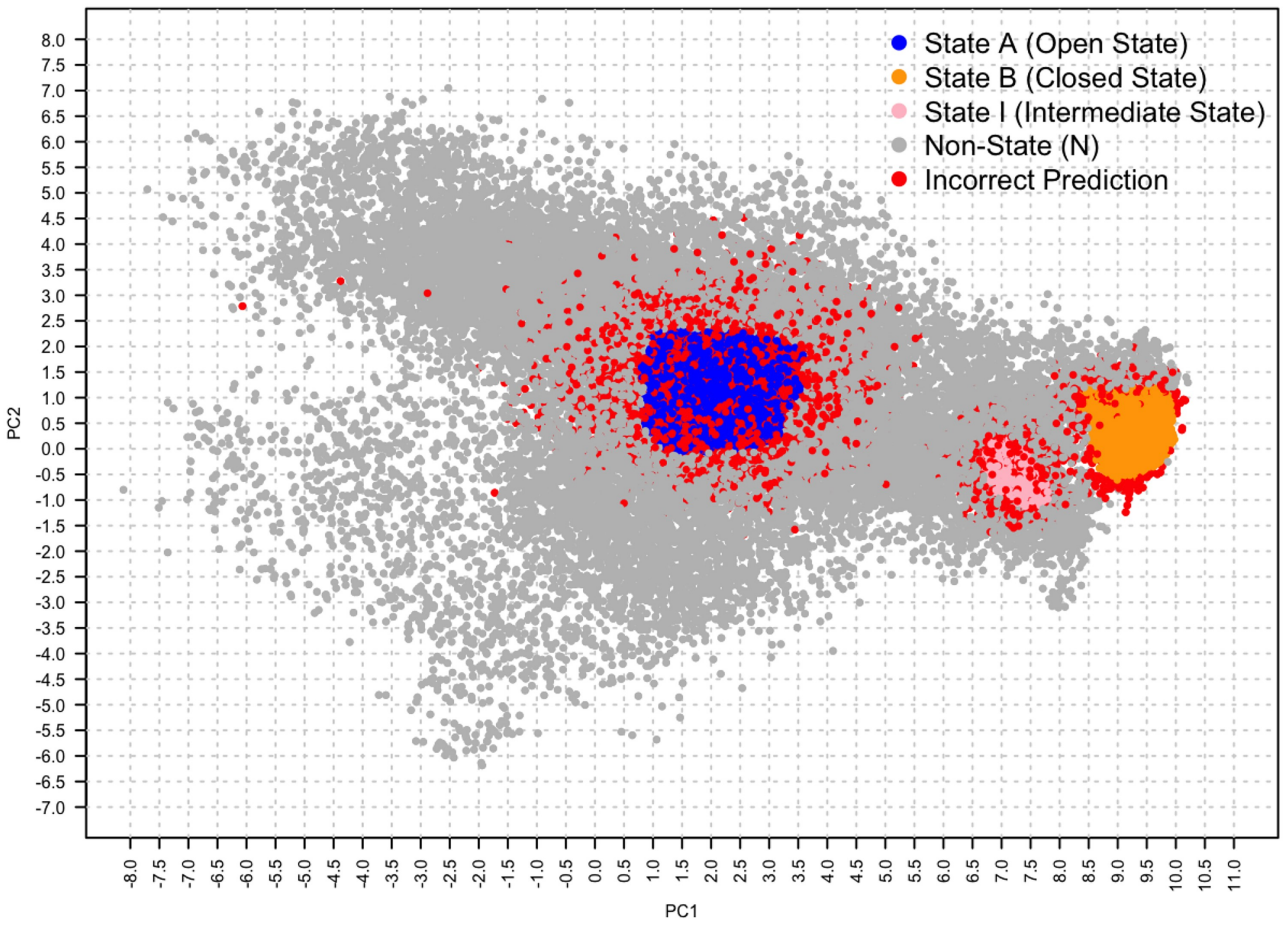
The PCA plot shows the neural network’s state predictions for 100 000 frames, using color coding to distinguish between ADK states. Red dots indicate incorrect predictions, while blue, pink, and grey dots represent correct predictions for the open (A), intermediate (I), and non-state (N), respectively.

Similar to Class B, Class N was predicted with high accuracy across most models. LGBM demonstrated the best performance at smaller trajectory sizes, achieving an accuracy of 0.92 for 5000 frames. For larger trajectory sizes (50 000 frames), Neural Networks achieved an accuracy of 0.91, showcasing their adaptability with increased data and capacity to capture complex, distributed patterns. A projection of the correctness of predictions on the (PC1, PC2) space for LR and NN is reported in Figs 4 and 5, respectively, on the 100 000-frame dataset with GraphSage embeddings, where ADK states are colour-coded: blue dots for the open state (A), pink for the intermediate state (I), orange for the close state (B), and grey for the non-state (N), while incorrect predictions are shown as red dots.

An overview of the performance across dataset sizes (see Table 1) suggests that 50 000 frames is the optimal size, offering the highest balanced accuracy across protein states. This dataset size is consistent with the sampling of conformations every 20 ps. This timescale aligns with loop rearrangements and small domain motions underpinning larger conformational changes. This dataset size represents a good compromise for training on trajectories of up to the μs scale.

## 4 Conclusion

In this study, we introduced MDGraphEmb, the first open-source, domain-specific toolkit for encoding protein conformational dynamics from protein MD simulations into graph embeddings suitable for machine learning. Unlike general-purpose frameworks such as PyG and DGL, MDGraphEmb provides a complete and tailored workflow, from MD trajectory preprocessing to graph construction, embedding generation, and classification. It integrates multiple embedding methods (Node2Vec, GCN, GAT, and GraphSAGE) and various machine learning models.

We addressed the core challenge of compressing high-dimensional structural dynamics into informative representations by encoding and compressing information in graph models to enhance the signal-to-noise ratio and transform molecular dynamics data into a tabular format suitable for effective machine learning predictions. By systematically comparing graph embedding methods, we evaluated how well each approach preserved signal relevant to protein state classification. Among them, GraphSAGE offered the best trade-off between expressiveness and scalability, particularly for large datasets. We showed how supervised models can be trained to predict frame-level properties on unseen data. We demonstrated this by classifying the conformational states of the ADK protein, which exhibits distinct functional transitions, including intermediate and transient states between experimentally characterized open and closed conformations. To evaluate the generalizability of the approach, MDGraphEmb was also tested on two structurally and dynamically diverse systems: PlnE, an α-helical antimicrobial peptide with a high degree of conformational flexibility, and HIV-1 protease, which undergoes large-scale loop opening and closing motions that regulate access to its active site. These contrasting systems demonstrate that MDGraphEmb is suitable for proteins with a spectrum of dynamics changes, different structural classes and functional mechanisms.

While clustering remains a useful exploratory tool, it struggles to capture continuous transitions and generalize across simulations. The supervised learning approach presented here addresses these limitations by learning from labelled examples, enabling robust and scalable prediction of conformational states in new datasets.

The MDGraphEmb library can be readily extended to study and characterize long MD simulations. Additionally, it may serve as a valuable tool for investigating the impact of mutations on the intrinsic dynamics of proteins by comparing different state samples. Overall, MDGraphEmb lowers the barrier to applying graph learning in molecular simulations and enables scalable, reproducible, and biologically meaningful analysis of protein dynamics. It offers a practical foundation for applications such as mutation impact analysis, long-timescale trajectory annotation, and automated state classification in high-throughput workflows.

## Supplementary Material

btaf420_Supplementary_Data

## Data Availability

Relevant data underpinning this publication can be accessed from Brunel University London’s data repository under CC BY licence: https://doi.org/10.17633/rd.brunel.c.7664645.
