# Supplementary material for: MDGraphEmb: a toolkit for graph embedding and classification of protein conformational ensembles
Source: Bioinformatics. 2025 Jul 31;41(9):btaf420. doi: 10.1093/bioinformatics/btaf420 (PMC12453676; doi:10.1093/bioinformatics/btaf420)
Supplement: btaf420_Supplementary_Data [file btaf420_supplementary_data.pdf]

Journal Title Here, 2024, pp. 1–10

doi: DOI HERE
Advance Access Publication Date: Day Month Year
Paper

PAPER

MDGraphEmb: A Toolkit for Graph Embedding and Classification of Protein Conformational Ensembles

Ferdoos Hossein Nezhad,[1] * Namir Oues,[1] Massimiliano Meli[2] and Alessandro Pandini[1,3] *

[1]Department of Computer Science, Brunel University of London, Kingston Lane, UB8 3PH, Uxbridge, UK, [2]Istituto di Scienze e Tecnologie Chimiche ''Giulio Natta" – SCITEC, Consiglio Nazionale delle Ricerche, Via Mario Bianco 9, 20131, Milano, Italy and [3]The Thomas Young Centre for Theory and Simulation of Materials, SW7 2AZ, London, UK

*Corresponding authors: Ferdoos.HosseinNezhad@brunel.ac.uk Alessandro.Pandini@brunel.ac.uk

FOR PUBLISHER ONLY Received on Date Month Year; revised on Date Month Year; accepted on Date Month Year

Abstract

The following content includes Supplementary Materials with detailed information on the workflow steps, corresponding GitHub scripts, the architectures of the graph embedding methods, the parameters used for embedding and machine learning, the implementation of machine learning models within the MDGraphEmb toolkit, as well as details of the three case studies, including system preparation, simulation and evaluation results.

Supplementary Materials

Methods

Workflow Steps

In the first step of the MDGraphEMB pipeline, the protein simulation trajectory and topology files are processed to generate graph-based representations of protein structures, where nodes correspond to protein Cα atoms and edges encode spatial proximity based on a distance threshold. GraphSAGE (Graph Sample and Aggregate)(1) is then applied to these graphs to produce node embeddings, capturing local structural information within a low-dimensional feature space. GraphSAGE is the recommended embedding method due to its ability to efficiently handle large dynamic datasets, generalise inductively to unseen nodes, and balance computational scalability with high predictive performance. Nevertheless, the MDGraphEMB toolkit also provides support for alternative embedding techniques such as Node2Vec (2), which captures community structure via random walks, Graph Convolutional Networks (GCN) (3), which apply convolution operations over graph neighbourhoods, and Graph Attention Networks (GAT) (4), which assign adaptive attention weights to neighbouring nodes. While these methods are available, GraphSAGE remains the suggested option for its robustness and suitability to encode protein dynamics (Figure 1a).

In the second step the embeddings in tabular format are joined with the target variable describing the conformational state of each frame, creating a dataset for supervised learning tasks. This step associate each embedding vector with its corresponding protein label based on frame indices extracted from the trajectory. The resulting dataset is a matrix wherein each row corresponds to a protein conformation described by its learned embedding features and an associated protein functional class label (Figure 1b). In cases where users aim solely to generate graph embeddings from molecular dynamics (MD) data, the `target.dat` file is not required. This file, along with the second step of the workflow, is only necessary for supervised machine learning tasks where labelled target data is used for training and model evaluation.

In the third step, the merged dataset is used to train machine learning models to predict the conformational state of each protein structure (Figure 1c). MDGraphEMB provides a default set of classifiers including Logistic Regression, Random Forest, XGBoost, and LightGBM, selected for their reliability, scalability, and efficient performance on large datasets. In addition to these models, users may choose to employ other classifiers integrated into the toolkit, such as Neural Networks (NN), Convolutional Neural Networks (CNN), and Support Vector Machines (SVM), which are particularly useful when deeper representation learning is desired and runtime constraints are less critical. Classification performance is evaluated using multiple metrics, including overall accuracy, class-specific accuracy, precision and recall, confusion matrices, precision-recall curves, ROC curves, confusion matrix. Predictions on a test set can be generated for all machine learning models and the exact frames used for training and testing can be recorded for reference. A report can be generated showing a comprehensive analysis of model performance across different conformational states (Figure 1d).

A complete tutorial script is provided in the GitHub repository to demonstrate the full pipeline workflow, running sequentially from embedding generation to model evaluation. This minimal example is designed to be user-friendly and easily

© The Author 2024. Published by Oxford University Press. All rights reserved. For permissions, please e-mail: journals.permissions@oup.com

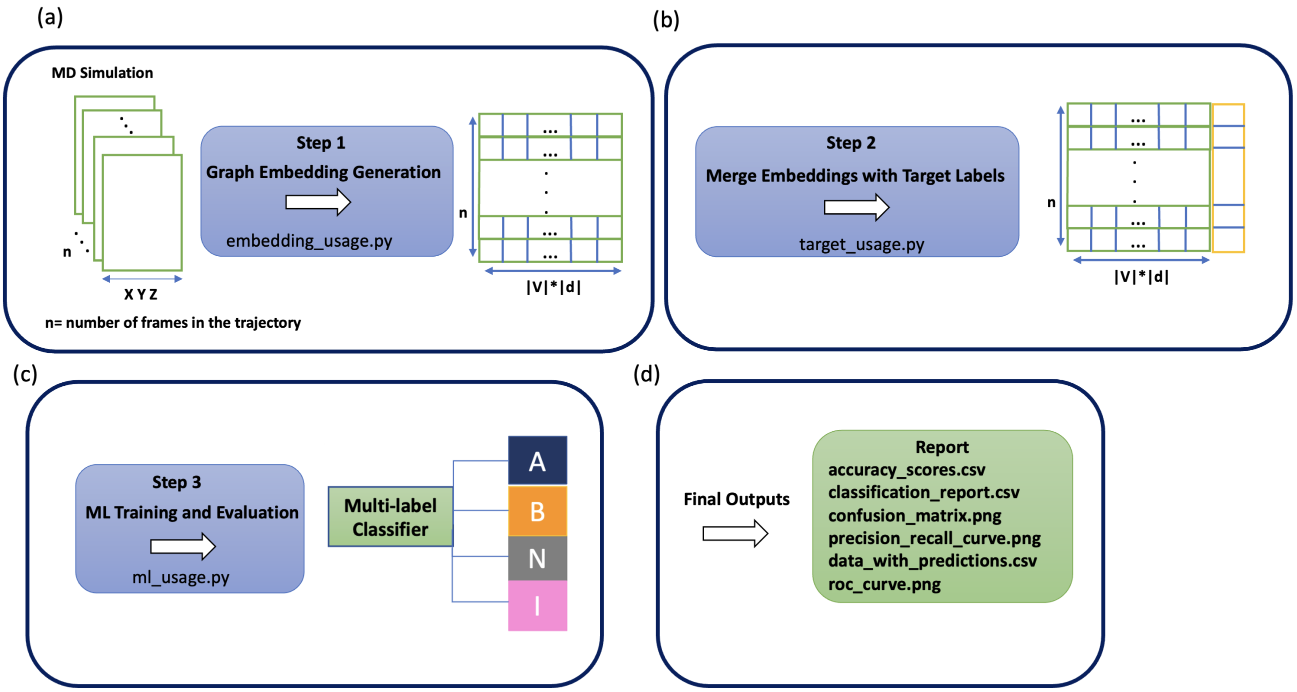

Fig. 1. Extended workflow of the **MDGraphEMB** toolkit with corresponding Python scripts associated with each step. Step 1: Embedding Generation (`embedding_usage.py`), Step 2: Merge with Targets (`target_usage.py`), Step 3: ML Training and Evaluation (`ml_usage.py`). The final report directory contains overall model accuracy, per-class accuracy, precision, recall, F1-score, support for each class, confusion matrices, precision-recall curves, ROC curves, and data with predictions generated by all machine learning models, including the exact frames used for training and testing.

Table 1. Overview of the MDGraphEMB pipeline steps, associated scripts and corresponding GitHub links.

Pipeline Step	Script Name	Description	GitHub Link
Step 1: Embedding Generation	`embedding_usage.py`	Generates GraphSAGE embeddings from MD trajectory data. GraphSAGE is the recommended embedding method; however, Node2Vec, GCN, and GAT are also available.	View on GitHub
Step 2: Merging embeddings with target	`target_usage.py`	Merges the generated embeddings with a target variable of conformational state labels for supervised learning.	View on GitHub
Step 3: ML Training & Evaluation	`ml_usage.py`	Trains multiple machine learning classifiers (e.g., Logistic Regression, Random Forest, XGBoost, LightGBM). Additional classifiers such as Neural Network, CNN, and SVM are available if runtime allows.	View on GitHub
Full Pipeline Example	`tutorialexample.py`	A complete walkthrough that runs all steps sequentially, providing a minimal working example.	View on GitHub

modifiable, allowing users to adapt the pipeline to different embedding methods, target files, or classification strategies depending on their scientific objectives (Table 1).

Architectures of the Embedding Methods

This section describes in detail the architectures of the embedding methods implemented in the MDGraphEMB toolkit.

The GraphSAGE model generates node embeddings by sampling and aggregating features from a node's local neighbourhood. The input consists of a node feature matrix and an edge index, where each node has associated features, such as degree information. The model is composed of two SAGEConv layers. The first layer aggregates features from neighbouring nodes using mean aggregation, while the second layer refines these hidden representations. Each layer has 64 hidden channels, and the final output layer produces 64-dimensional embeddings. ReLU activation functions are applied after each layer, except the last one. This two-layer configuration balances computational efficiency and model depth, ensuring that nodes can effectively aggregate information from their neighbours without adding unnecessary complexity. The embeddings generated after the final layer are ready for downstream tasks.

The GCN (Graph Convolutional Network) model learns node embeddings by propagating node feature information across graph layers via convolutions. This implementation includes four layers of GCNConv, each with 64 hidden channels.

Batch normalization and ReLU activation functions are applied after each convolution, except for the last layer. Dropout rates of 0.5 are used to prevent over-fitting. The final layer produces 64-dimensional embeddings for each node. The model is trained using a learning rate of 0.01 over 200 epochs, with gradient clipping set at 5.0 to stabilize training.

The Node2Vec model generates node embeddings by simulating random walks on the graph and applying the Word2Vec model to the sequences of nodes encountered. Each node is treated as a "word," and the embeddings are learned based on node co-occurrence in the random walks. In this configuration, each node undergoes 10 random walks of length 80. The resulting embeddings have a dimensionality of 64, with a context window of size 10. This approach captures both the local and global structure of the graph by balancing breadth-first (BFS) and depth-first (DFS) search strategies during random walks.

The GAT model incorporates attention mechanisms to learn node embeddings by weighting the importance of neighbouring nodes. The model uses four layers of GATConv with multi-head attention. The first three layers have 8 attention heads, while the final layer has 1 attention head. Each layer has 64 hidden channels, and the final output layer produces 64-dimensional embeddings. Dropout rates of 0.6 are applied after each attention layer, and ReLU activation functions are used after each layer except the last. This attention mechanism allows the model to focus on the most relevant neighbours, improving the quality of the learned embeddings. Refer to (Table 5) for the configurations of the graph embedding methods.

Machine Learning Models

In *MDGraphEmb*, several machine learning models are implemented to classify protein states, including open, closed, intermediate and undefined (none) state. These models are designed to handle multi-class classification tasks and have been configured to manage potential class imbalances inherent in protein datasets. The Logistic Regression model (5) is a simple yet powerful classification method. It uses the `class_weight='balanced'` parameter to account for imbalances in the dataset by adjusting the weight of each class, ensuring that less frequent states such as "intermediate" are not under-represented during training. The model is optimized with a maximum of 1000 iterations (`max_iter=1000`), ensuring convergence in the learning process even with more complex data distributions. The Random Forest model (5) is an ensemble learning method built on multiple decision trees and predict the class label by majority vote on the label predicted by individual trees. Similar to logistic regression, the model uses `class_weight='balanced'` to adjust for class imbalances. This ensures that each protein state is equally weighted during training, improving model generalization across the dataset. The XGBoost model (7), a gradient-boosting decision tree algorithm, is employed for its high performance and flexibility in handling complex and imbalanced datasets. In this study, the model is configured with `max_depth=3`, `n_estimators=80` and uses the `hist` tree method for faster histogram-based training. Parallel computation is enabled with `n_jobs=2`, and verbosity is set to `1` to monitor the training process. These parameters balance model complexity and computational efficiency while preserving the ability to capture subtle distinctions between conformational states. The LightGBM model (8), another gradient-boosting decision tree method optimised for speed and scalability, is also available in MDGraphEmb. In this implementation, `class_weight='balanced'` is used to address class imbalance and the model is configured with `max_depth=3`, `num_leaves=7`, `n_estimators=80`, and `force_col_wise=True` to ensure consistent performance with the dataset structure. Similar to XGBoost, `n_jobs=2` and `verbosity=1` are set to optimise training speed and provide feedback during execution. These settings enable LightGBM to effectively model high-dimensional protein embedding data while improving classification accuracy for under-represented classes. The SVM model (5) identifies the hyperplane that best separates the different classes. The parameter `class_weight='balanced'` is used to handle imbalances in the dataset. While the parameter `probability=True`enables the output of probability estimating the confidence for each protein state prediction. This feature is especially valuable in scenarios where certain states (e.g., intermediate) may overlap with others, and precise probability estimates help differentiate between them. The Neural Network (NN (6)) model implemented in *MDGraphEmb* is a fully connected feed-forward neural network designed for multi-class classification tasks. The network consists of three layers: the first layer has 256 units with `ReLU` activation, followed by BatchNormalization and Dropout (0.5); the second layer has 128 units with similar settings; and the final layer is a softmax layer with output units corresponding to the number of protein states. The model is optimised using the Adam optimiser with a learning rate of 0.0005, and categorical cross-entropy is used as the loss function. The network is trained for 30 epochs with a batch size of 32, while EarlyStopping monitors validation loss to prevent overfitting. This deep learning model captures complex, non-linear relationships between the features defining each protein state.The CNN model (6) includes two Conv1D layers: the first with 64 filters and a kernel size of 3 and the second with 128 filters and the same kernel size. Both layers use `ReLU` activation function and are followed by MaxPooling with a pool size of 2. The flattened output is passed through a Dense layer with 128 units and Dropout (0.5) before being classified through a softmax output layer. The CNN model is trained using categorical cross-entropy with 30 epochs and a batch size of 32. EarlyStopping is applied to monitor validation loss, ensuring that the model does not overfit. For the configurations of the machine learning models, refer to (Table 6).

Train-Test Set Splitting and Evaluation Strategy

Each ML model is trained to correctly classify protein state label from protein structures of each frame in the simulation. The input is protein embedding data, provided in `.csv` format; this input is first loaded and preprocessed to ensure proper feature encoding and target assignment. The dataset is partitioned into training and testing subsets using the `split_data()` function, with 70% allocated to the training set and 30% to the testing set (`test_size=0.3`). Stratified sampling ensures that the distribution of protein states is preserved across both subsets, while fixed random seeding (`random_state=50`) ensures consistency and reproducibility. Feature scaling is applied conditionally based on model-specific requirements. For models sensitive to feature scaling, such as NN, CNN, and SVM, the `StandardScaler` standardizes both the training and testing sets. The CNN model undergoes additional preprocessing, including input reshaping to match architectural requirements. This pipeline offers both shallow models, such as Logistic Regression and Random Forest, and deep learning models, optimizing classification performance across diverse architectures. The evaluation is conducted on the independent

test set to ensure an unbiased assessment of generalization. Key performance metrics—including accuracy, precision, recall, and F1-score—are computed to assess classification performance across the four protein states. Confusion matrices, ROC curves, and precision-recall curves provide a comprehensive analysis of the model's ability to differentiate between states. By strictly maintaining the independence of the test set, the evaluation reflects the models' true generalization capability without any risk of data leakage. Following the evaluation, a `.csv` file is generated containing essential information about the partitioning of frames into training and test. The file includes columns for the original protein state, predicted state, and set type (train/test), ensuring traceability of the experimental process. This output supports post-experimental analyses, such as identifying misclassified frames and assessing model robustness across different regions of the dataset. The systematic saving of predictions and evaluation metrics within the `report` directory ensures reproducibility and facilitates further investigations into classification performance.

Case Study Systems: Preparation and Simulation

Case Study 1: System Preparation and Simulation of Adenylate Kinase

The Adenylate Kinase (ADK) structure (PDBID: 4AKE) was retrieved from the Protein Data Bank. A double mutant (V135G + V142G) was generated from this structure (PDB ID: 4AKE) using PyRosetta (9). Both wild-type and mutated structures were placed in a cubic simulation box with a minimum solute-box boundary distance of 10 Å. The system was solvated with TIP3P water molecules and four sodium ions (Na+) were added to neutralize the total charge. Energy minimization was conducted in three stages. The first step employed the steepest descent algorithm for 50,000 steps with positional restraints of 2000 kJ/mol/nm^2 applied to the heavy atoms of the protein, ensuring initial system relaxation while preserving the protein backbone structure. The second minimization step proceeded without positional restraints, allowing further structural relaxation. Finally, a conjugate gradient algorithm with flexible constraints was used to reach a convergence criterion where the maximum force was reduced to less than 10 kJ/mol/nm^2 over 10,000 steps.

Following minimisation, the system underwent equilibration in two phases: temperature equilibration under the NVT ensemble and pressure equilibration under the NPT ensemble. Temperature equilibration was performed in six sequential steps, gradually heating the system from 200 K to 300 K while applying positional restraints to maintain structural integrity. The first step raised the temperature to 200 K with positional restraints of 2000 kJ/mol/nm^2 on the heavy atoms, using the Berendsen thermostat for temperature coupling. The temperature was subsequently increased to 250 K with reduced restraints of 1000 kJ/mol/nm^2, then to 300 K under the same conditions. The final three steps progressively reduced the positional restraints to 500, 250, and finally 0 kJ/mol/nm^2, allowing complete relaxation at 300 K.

Pressure equilibration followed under the NPT ensemble in two stages. Initially, the system was equilibrated to 1 bar using the Berendsen barostat with positional restraints of 210 kJ/mol/nm^2 on the protein's heavy atoms for 500 ps. The second stage maintained the restraints while switching to the Parrinello-Rahman barostat for an additional 500 ps. The V-rescale thermostat was applied with a coupling constant of 0.1 ps to ensure accurate temperature control. This approach ensured the system reached thermal and pressure stability before production simulations.

Case Study 2: System Description of Plantaricin E

Plantaricin E (PlnE) is a small, amphipathic α-helical antimicrobial peptide produced by *Lactiplantibacillus plantarum*. It is part of a class IIb two-peptide bacteriocin system, acting in synergy with Plantaricin F (PlnF). Together, they consist of two peptides: the 33-residue PlnE and the 34-residue PlnF, both of which are required in approximately equimolar amounts to achieve maximal antimicrobial activity (10). The structure of PlnE was determined by NMR in the presence of DPC micelles (PDB ID: 2JUI), revealing its helical character and dynamic behaviour. These features make Plantaricin E a suitable test system for evaluating the ability of MDGraphEmb to detect localised conformational changes in flexible peptide structures. Peptides are known to exhibit significant conformational freedom in solution, making them excellent test cases for evaluating the sensitivity of graph-based representations such as those used in MDGraphEmb. See Figure 6 for the structural details of PlnE and visualisation of target labelling in the (PC1, PC2) space of PlnE.

Case Study 3: System Description of HIV-1 protease

HIV-1 protease plays an essential role in viral infection and replication by processing the polyproteins produced during the viral life cycle into the individual proteins required to assemble a mature virion (11). It is a homodimeric aspartyl protease, with its active site located at the interface of the two subunits along a twofold axis of symmetry. The substrate-binding cleft runs perpendicular to this axis and is covered by loop regions (residues 45–54 in each monomer) that act as flexible flaps or gates regulating access to the catalytic centre (see Figure 9). Although the conformational change associated with flap motion is relatively small, it is functionally critical for substrate recognition and catalysis. This subtle yet essential movement provides an ideal test case for evaluating MDGraphEmb's ability to capture and classify small conformational changes. See Figure 9 for for the structural details of HIV-1 protease and visualisation of target labelling in the (PC1, PC2) space of HIV-1 protease.

System Preparation and Simulation of PlnE and HIV-1 protease

The structures of HIV-1 protease and PlnE were obtained from the RCSB PDB database (PDB IDs 2PC0 and 2JUI respectively). For both systems, crystallographic water molecules and any organic ligands used during experimental structure determination were removed prior to simulation. All MD simulations — both standard and enhanced-sampling — were carried out using the AMBER 22 simulation package (12), with the ff14SB force field (13) and TIP3P water model (14). Simulations were accelerated using the CUDA implementation for GPUs. The systems were placed in truncated octahedral boxes with a minimum distance of 14 Å between any protein atom and the box edge. Solvation was performed using TIP3P water molecules, and Na^+ and Cl^- ions were added to neutralise the system charge. Energy minimisation was performed in two stages: 5,000 steps of steepest descent followed by 5,000 steps of

conjugate gradient minimisation. The minimised systems were then equilibrated for 10 ns at 300 K using Langevin dynamics with a collision frequency of 1 ps^{-1}. Production simulations were run in the NPT ensemble at 1 atm, using the Berendsen barostat (15). Electrostatic interactions were treated with the Particle Mesh Ewald method (16), and SHAKE constraints (17) were applied to all covalent bonds involving hydrogen atoms. A 2-fs time step was used for all simulations, with a 10 Å cutoff for van der Waals interactions.

A preliminary MD simulation was performed on the apo wild-type HIV-1 protease in its open conformation (PDB ID: 2PC0). The Principal Component Analysis (PCA) of the trajectory revealed that the first two mode of motions correspond to the gate-opening and gate-closing motions reported in previous studies (18). These two components were then used as collective variables in a metadynamics simulation (using PLUMED (19)) aimed at enhancing sampling of the conformational space associated with flap dynamics (18). This approach enabled exploration of the free energy landscape associated with the structural transitions controlling access to the active site. The production run was of 500ns saving a frame every 10ps for downstream embedding and classification analysis.

For the plnE system, an initial exploratory 200 ns MD simulation in aqueous solution - conducted in the absence of DPC micelles. The α-helical conformation observed in the NMR structure was found to be unstable. Notably, conformational deviations were recorded as early as the equilibration phase, which is consistent with the expected structural response to changes in environmental conditions. This last conformation was used as starting point of an enhanced MD simulation (using PLUMED (19)) with the aim to explore the conformational freedom of plnE. The free energy landscape of plnE was explored using a collective variable built as combination of the backbone PHI and PSI peptide from residue 3 to residue 27.

Results

Different embeddings were generated from frames of the protein dynamics simulations using GAT, GCN, GraphSAGE, and Node2Vec for frame sizes of 5,000, 10,000, and 25,000. Various machine learning models were implemented on these embeddings to classify conformational states across different frame sizes. We investigated three key aspects: embedding methods, machine learning models, and dataset sizes, to identify the optimal combination of graph embedding and machine learning for protein state classification. A detailed comparison of the embedding techniques across various machine learning algorithms is presented in Tables 2, 3, and 4, covering frame sizes of 5,000, 10,000, and 25,000. The results demonstrate that GraphSAGE consistently produces high-quality embeddings, leading to superior and more stable classification performance compared to other methods. See Figures 2, 3, and 4 for a clearer comparison of accuracy by class, machine learning model and embedding method across different frame sizes. To further investigate scalability, we trained the machine learning models on larger datasets using only the GraphSAGE embeddings with frame sizes of 50,000 and 100,000. Figure 5 presents the class-specific accuracy across models and frame sizes using GraphSAGE. For a detailed evaluation of different machine learning metrics by class for the ADK system on 50,000 frames—the best-performing dataset size — refer to Table 7. A comparison of class-specific and overall accuracy across different trajectory sizes using various machine learning models with GraphSAGE embeddings for the second system, PlnE, is reported in Table 8. The results indicate that most ML models perform well on this system in accurately classifying states A and B, demonstrating the effectiveness of the learned embeddings in capturing the distinct features of these conformational states. Table 9 presents a comparison of class-wise performance metrics for various machine learning models using the GraphSAGE embedding method on 70,000 frames, for the second system, PlnE. Figures 6 and 7 show the performance of the two top-performing machine learning NN and SVM models for the PlnE system. A comparison of class-specific and overall accuracy across different trajectory sizes using various machine learning models with GraphSAGE embeddings for the third system, HIV-1 Protease, is presented in Table 10. The results indicate that the NN and LightGBM models perform particularly well in classifying states A (open) and B (closed) with high accuracy. Table 11 provides a detailed comparison of class-wise performance metrics for these models using GraphSAGE embeddings on 50,000 frames. Figures 10 and 11 illustrate the performance of the two top-performing models, NN and LightGBM, highlighting their robustness in distinguishing conformational states within the HIV-1 Protease system. Overall, across all machine learning models and systems (ADK, PlnE, and HIV-1 protease), neural networks consistently achieved better performance in identifying conformational states, including under-represented or transitional states. This highlights its effectiveness in learning complex representations from graph-based embeddings of protein dynamics.

References

1. Will Hamilton, Zhitao Ying, and Jure Leskovec. Inductive representation learning on large graphs. *Advances in Neural Information Processing Systems*, 30, 2017.
2. Aditya Grover and Jure Leskovec. Node2vec: Scalable feature learning for networks. In *Proceedings of the 22nd ACM SIGKDD International Conference on Knowledge Discovery and Data Mining*, pages 855–864, 2016.
3. Thomas N Kipf and Max Welling. Semi-supervised classification with graph convolutional networks. *arXiv preprint arXiv:1609.02907*, 2016.
4. Petar Veličković, Rex Ying, Matilde Padovano, Raia Hadsell, and Charles Blundell. Neural execution of graph algorithms. *arXiv preprint arXiv:1910.10593*, 2019.
5. F. Pedregosa, G. Varoquaux, A. Gramfort, V. Michel, B. Thirion, O. Grisel, M. Blondel, P. Prettenhofer, R. Weiss, V. Dubourg, J. Vanderplas, A. Passos, D. Cournapeau, M. Brucher, M. Perrot, and É. Duchesnay. Scikit-learn: Machine learning in Python. *Journal of Machine Learning Research*, 12:2825–2830, 2011.
6. Martín Abadi, Ashish Agarwal, Paul Barham, Eugene Brevdo, Zhifeng Chen, Craig Citro, Greg S. Corrado, Andy Davis, Jeffrey Dean, Matthieu Devin, Sanjay Ghemawat, Ian Goodfellow, Andrew Harp, Geoffrey Irving, Michael Isard, Yangqing Jia, Rafal Józefowicz, Lukasz Kaiser, Manjunath Kudlur, Josh Levenberg, Dan Mane, Rajat Monga, Sherry Moore, Derek Murray, Chris Olah, Mike Schuster, Jonathon Shlens, Benoit Steiner, Ilya Sutskever, Kunal Talwar, Paul Tucker, Vincent Vanhoucke, Vijay Vasudevan, Fernanda Viégas, Oriol Vinyals, Pete Warden, Martin Wattenberg, Martin Wicke, Yuan Yu, and Xiaoqiang Zheng. TensorFlow: Large-scale machine learning on heterogeneous systems. *Software available from tensorflow.org*, 2015.
7. Tianqi Chen and Carlos Guestrin. XGBoost: A scalable tree boosting system. In *Proceedings of the 22nd ACM SIGKDD International Conference on Knowledge Discovery and Data Mining*, pages 785–794, 2016.
8. Guolin Ke, Qi Meng, Thomas Finley, Taifeng Wang, Wei Chen, Weidong Ma, Qiwei Ye, and Tie-Yan Liu. LightGBM: A highly efficient gradient boosting decision tree. *Advances in Neural Information Processing Systems*, 30, 2017.
9. Jeffrey J. Gray, Brian D. Weitzner, and Scott E. Boyken. PyRosetta: a script-based interface for implementing molecular modeling algorithms using Rosetta. *Bioinformatics*, 26(5):689–691, 2010.
10. Nina Fimland, Per Rogne, Gunnar Fimland, Jon Nissen-Meyer, and Per Eugen Kristiansen. Three-dimensional structure of the two peptides that constitute the two-peptide bacteriocin plantaricin EF. *Biochimica et Biophysica Acta (BBA) - Proteins and Proteomics*, 1784(11):1711–1719, 2008.
11. H. Heaslet, R. Rosenfeld, M. Giffin, Y. C. Lin, K. Tam, B. E. Torbett, and C. D. Stout. Conformational flexibility in the flap domains of ligand-free HIV protease. *Acta Crystallographica Section D: Biological Crystallography*, 63(8):866–875, 2007.

Table 2. Comparison of class-specific and overall accuracy of different machine learning models across different embedding methods (5000 frames) for ADK

Embedding	ML Model	Class A	Class B	Class I	Class N	Model Accuracy
**GraphSage**	Logistic Regression	**0.57**	0.95	0.43	0.74	0.74
	Random Forest	0.28	0.97	0.43	0.88	0.81
	XGBoost	0.27	0.97	**0.55**	0.90	**0.83**
	LightGBM	0.24	0.97	**0.55**	0.92	**0.83**
	Neural Network	0.47	**0.98**	0.48	0.81	0.78
	CNN	0.30	0.96	0.39	0.88	0.81
	Support Vector	0.31	0.97	0.30	0.89	0.82
**gcn**	Logistic Regression	0.30	0.91	0.36	0.82	0.76
	Random Forest	0.05	0.97	0.11	0.92	0.80
	XGBoost	0.18	0.96	0.50	0.85	0.78
	LightGBM	0.15	0.97	0.27	0.87	0.78
	Neural Network	0.25	0.97	0.27	0.86	0.79
	CNN	0.21	**0.98**	0.23	0.81	0.74
	Support Vector	0.03	0.96	0.02	0.94	0.81
**node2vec**	Logistic Regression	0.22	0.60	0.07	0.72	0.63
	Random Forest	0.06	0.55	0.00	0.91	0.73
	XGBoost	0.03	0.92	0.11	0.93	0.79
	LightGBM	0.01	0.83	0.07	0.96	0.80
	Neural Network	0.08	0.59	0.00	0.87	0.71
	CNN	0.06	0.51	0.00	0.90	0.72
	Support Vector	0.00	0.18	0.00	**0.99**	0.73
**gat**	Logistic Regression	0.47	0.88	**0.55**	0.71	0.70
	Random Forest	0.07	0.44	0.00	0.89	0.70
	XGBoost	0.17	0.86	0.16	0.85	0.75
	LightGBM	0.21	0.89	0.11	0.84	0.75
	Neural Network	0.05	0.42	0.00	0.88	0.69
	CNN	0.18	0.21	0.02	0.82	0.63
	Support Vector	0.24	0.30	0.09	0.59	0.50

Table 3. Comparison of class-specific and overall accuracy of different machine learning models across different embedding methods (10000 frames) for ADK.

Embedding	ML Model	Class A	Class B	Class I	Class N	Model Accuracy
**GraphSage**	Logistic Regression	**0.62**	0.95	0.63	0.73	0.75
	Random Forest	0.39	**0.98**	0.54	0.88	0.82
	XGBoost	0.28	**0.98**	0.58	0.91	0.82
	LightGBM	0.28	**0.98**	0.53	0.90	0.82
	Neural Network	0.47	0.97	**0.70**	0.85	0.81
	CNN	0.41	0.97	0.47	0.85	0.80
	Support Vector	0.34	**0.98**	0.41	0.91	**0.83**
**gcn**	Logistic Regression	0.32	0.86	0.40	0.81	0.74
	Random Forest	0.09	0.95	0.16	0.93	0.79
	XGBoost	0.19	0.95	0.41	0.87	0.77
	LightGBM	0.16	0.96	0.30	0.88	0.77
	Neural Network	0.27	0.97	0.31	0.85	0.77
	CNN	0.14	0.90	0.31	0.91	0.78
	Support Vector	0.08	0.94	0.03	0.94	0.79
**node2vec**	Logistic Regression	0.25	0.64	0.17	0.72	0.62
	Random Forest	0.04	0.60	0.02	0.92	0.72
	XGBoost	0.05	0.93	0.12	0.92	0.77
	LightGBM	0.04	0.92	0.07	0.93	0.78
	Neural Network	0.12	0.71	0.06	0.83	0.69
	CNN	0.06	0.69	0.09	0.91	0.73
	Support Vector	0.00	0.49	0.00	**0.98**	0.74
**gat**	Logistic Regression	0.18	0.18	0.26	0.31	0.27
	Random Forest	0.08	0.08	0.07	0.80	0.57
	XGBoost	0.07	0.10	0.03	0.78	0.56
	LightGBM	0.10	0.13	0.05	0.74	0.54
	Neural Network	0.13	0.11	0.00	0.72	0.53
	CNN	0.14	0.20	0.02	0.67	0.51
	Support Vector	0.08	0.11	0.02	0.78	0.56

Table 4. Comparison of class-specific and overall accuracy of different machine learning models across different embedding methods (25000 frames) for ADK.

Embedding	ML Model	Class A	Class B	Class I	Class N	Model Accuracy
**GraphSage**	Logistic Regression	**0.72**	0.96	**0.86**	0.69	0.74
	Random Forest	0.35	0.98	0.63	0.86	0.8
	XGBoost	0.28	0.97	0.69	0.91	**0.83**
	LightGBM	0.32	0.97	0.69	0.89	0.82
	Neural Network	0.58	0.98	0.77	0.82	0.81
	CNN	0.31	0.94	0.53	0.9	0.82
	Support Vector	0.4	0.97	0.63	0.89	**0.83**
**gcn**	Logistic Regression	0.48	0.93	0.56	0.74	0.73
	Random Forest	0.11	0.94	0.32	0.91	0.79
	XGBoost	0.23	0.96	0.55	0.85	0.78
	LightGBM	0.29	0.96	0.59	0.84	0.77
	Neural Network	0.38	0.96	0.61	0.78	0.75
	CNN	0.21	0.93	0.34	0.89	0.79
	Support Vector	0.14	0.95	0.21	0.92	0.79
**node2vec**	Logistic Regression	0.35	0.71	0.21	0.63	0.59
	Random Forest	0.04	0.71	0.04	0.91	0.74
	XGBoost	0.07	0.95	0.24	0.88	0.77
	LightGBM	0.08	0.94	0.3	0.89	0.77
	Neural Network	0.13	0.88	0.13	0.84	0.73
	CNN	0.01	**0.99**	0.0	0.88	0.75
	Support Vector	0.0	0.8	0.0	**0.96**	0.78
**gat**	Logistic Regression	0.22	0.18	0.27	0.33	0.29
	Random Forest	0.09	0.11	0.01	0.78	0.57
	XGBoost	0.11	0.14	0.05	0.71	0.53
	LightGBM	0.11	0.14	0.06	0.7	0.52
	Neural Network	0.13	0.15	0.01	0.7	0.53
	CNN	0.11	0.14	0.01	0.73	0.54
	Support Vector	0.1	0.11	0.02	0.76	0.55

Table 5. Graph Embedding Method Configuration.

Parameter Name	Description	Value(s)
`NUM_WALKS`	Number of random walks per node for Node2Vec embeddings.	10
`WALK_LENGTH`	Length of each random walk.	80
`EMBEDDING_SIZE`	Size of the embeddings (for Node2Vec).	64
`WINDOW`	Context window size for the word2vec model (used in Node2Vec).	10
`MIN_COUNT`	Minimum count of nodes in Node2Vec embeddings.	1
`BATCH_WORDS`	Batch size for processing words in Node2Vec.	4
`DIMENSIONS`	Dimensionality of the embeddings for node features.	16
`GraphSAGE Hidden Layers`	Number of hidden channels for the GraphSAGE layers.	64
`GraphSAGE Out Layers`	Number of output channels for the GraphSAGE layers.	64
`GraphSAGE Num Layers`	Number of layers in the GraphSAGE network.	2
`GCN Hidden Channels`	Number of hidden channels in the GCN network.	64
`GCN Output Channels`	Number of output channels in the GCN network.	64
`GCN Dropout Rates`	Dropout rates for each layer in GCN to avoid overfitting.	[0.5, 0.5, 0.5]
`GCN Learning Rates`	Learning rate for GCN training.	[0.01, 0.01, 0.01]
`GCN Num Epochs`	Number of epochs for GCN training.	200
`GCN Gradient Clip Norm`	Max norm value to clip gradients during backpropagation in GCN training.	5.0
`GAT Hidden Channels`	Number of hidden channels in the GAT layers.	64
`GAT Output Channels`	Number of output channels in the GAT layers.	64
`GAT Dropout Rates`	Dropout rates for each GAT layer to avoid overfitting.	[0.6, 0.6, 0.6, 0.6]
`GAT Learning Rates`	Learning rate for GAT training.	[0.005]
`GAT Num Epochs`	Number of epochs for GAT training.	200
`GAT Gradient Clip Norm`	Max norm value to clip gradients during backpropagation in GAT training.	5.0
`GAT Num Heads`	Number of attention heads per layer in the GAT model.	[8, 8, 8, 1]

Note: This table presents the configuration parameters for various graph embedding methods, including Node2Vec, GraphSAGE, GCN, and GAT.

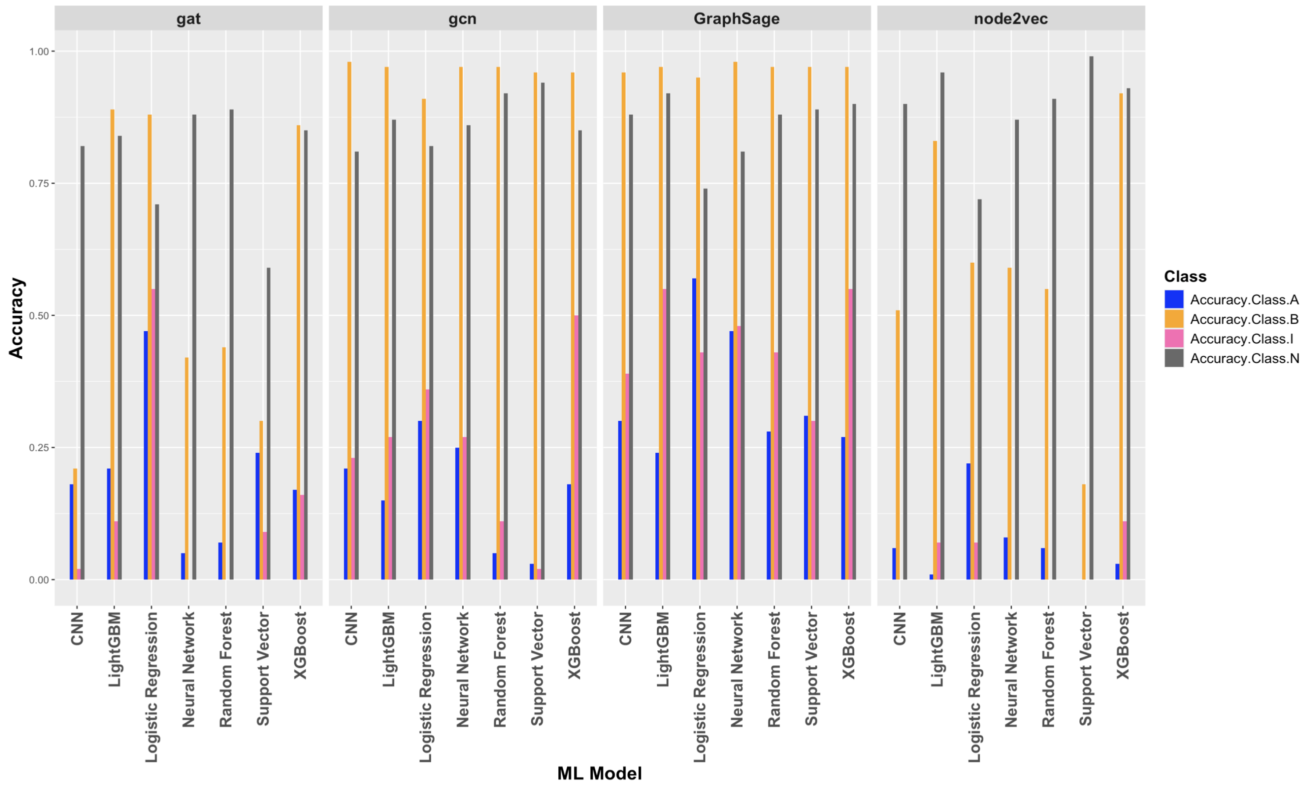

Fig. 2. Histogram Plot of Accuracy Comparison of All Embedding Methods Against Machine Learning Models Across All Classes for 5000 Frames for ADK.

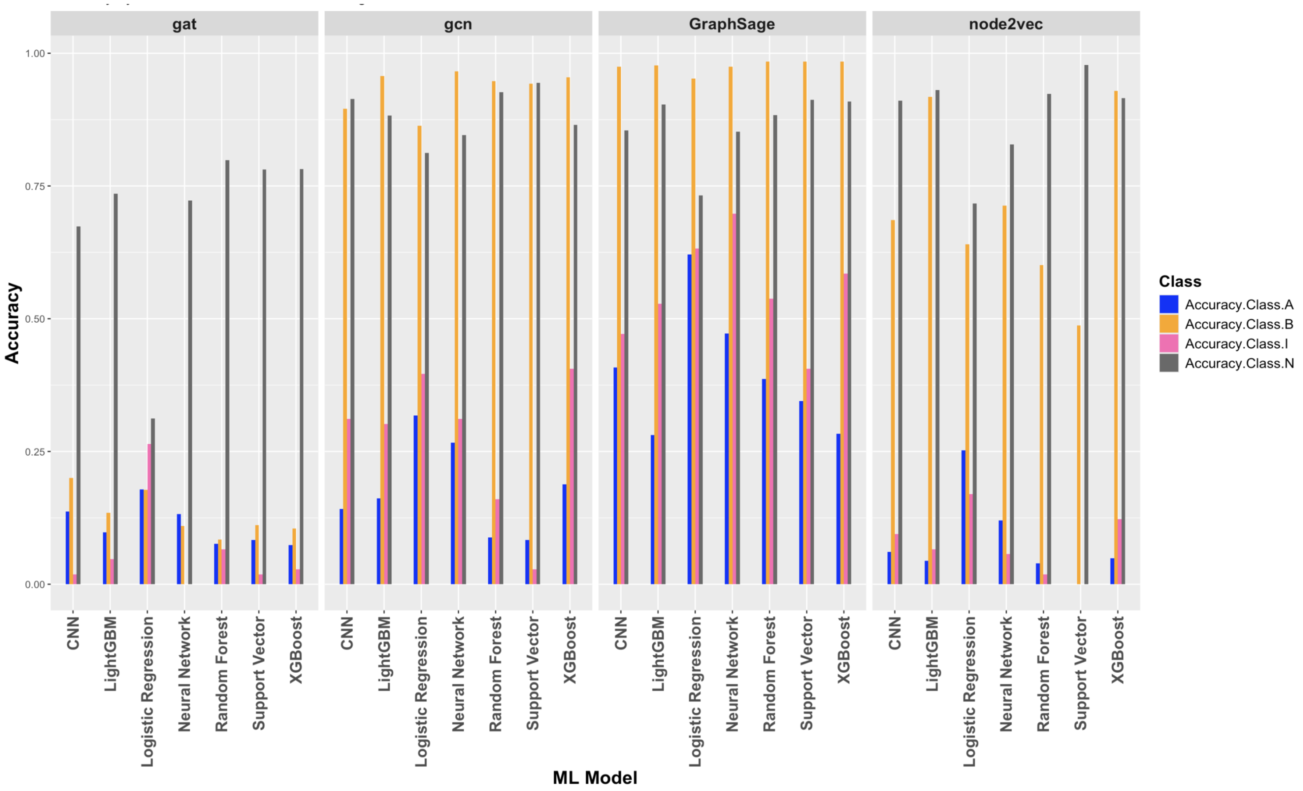

Fig. 3. Histogram Plot of Accuracy Comparison of All Embedding Methods Against Machine Learning Models Across All Classes for 10000 Frames for ADK.

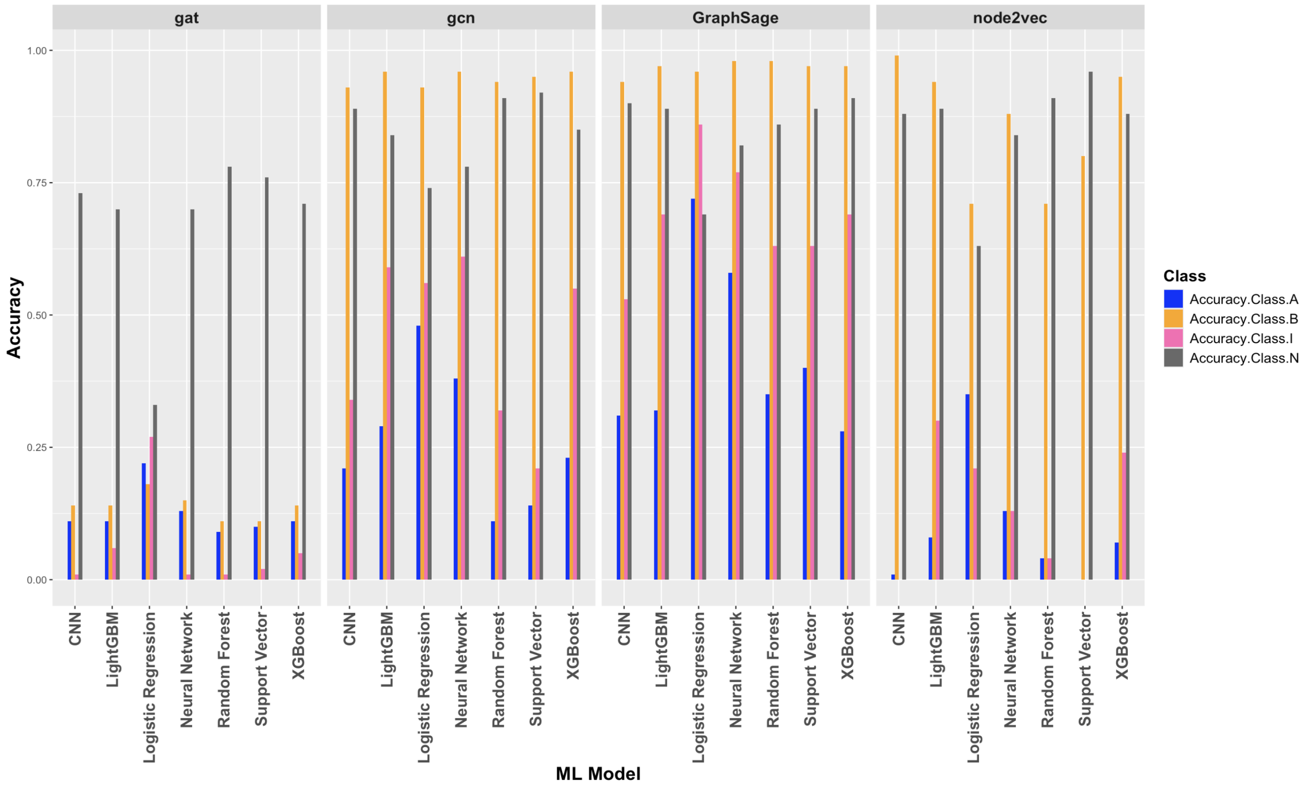

Fig. 4. Histogram Plot of Accuracy Comparison of All Embedding Methods Against Machine Learning Models Across All Classes for 25000 Frames for the ADK.

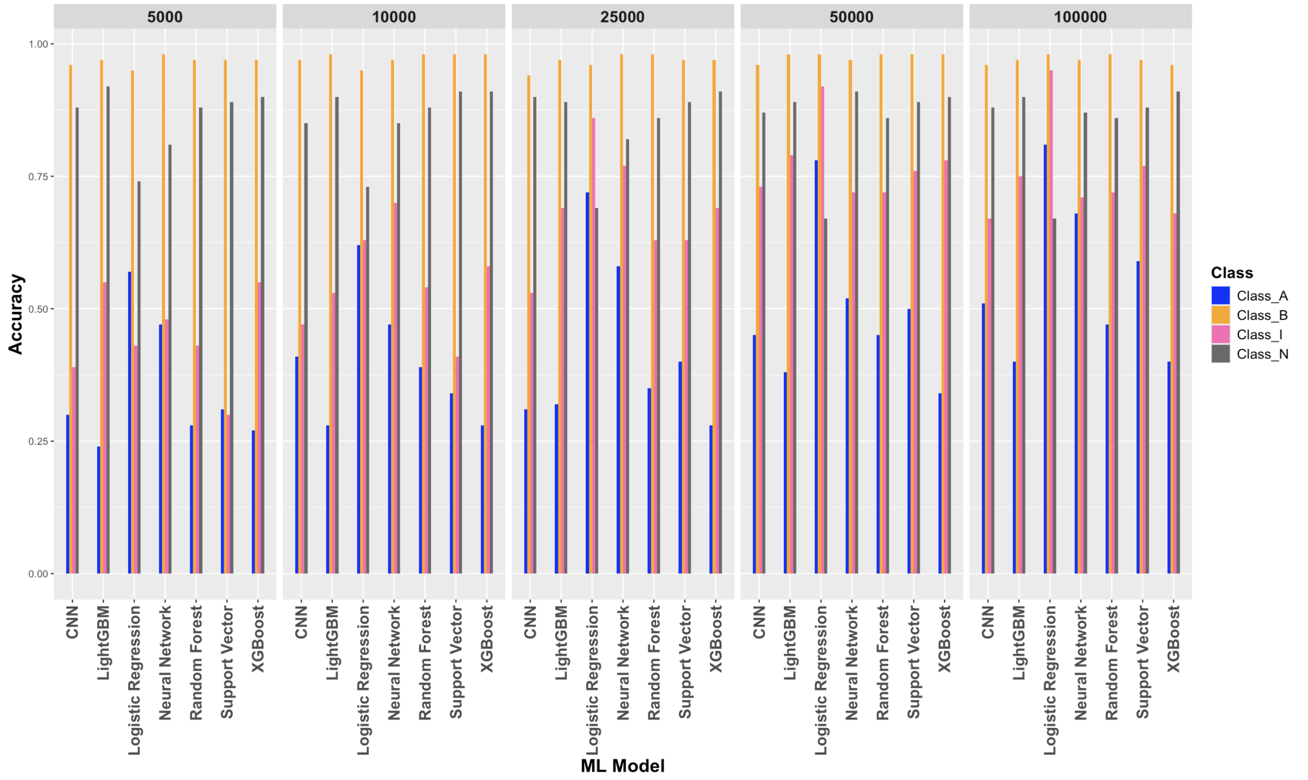

Fig. 5. Histogram Plot of Class-Specific Accuracy Across ML Models and Frames for GraphSage Embedding for ADK.

Table 6. Configuration Table of Machine Learning Models.

Model Name	Key Parameters
**Logistic Regression**	- `class_weight='balanced'`: Handles class imbalance by adjusting weights.<br>- `max_iter=1000`: Maximum number of iterations for optimization.
**Random Forest**	- `class_weight='balanced'`: Adjusts weights for imbalanced classes.
**XGBoost**	- `max_depth=3`: Limits tree depth to prevent overfitting.<br>- `n_estimators=80`: Number of boosting rounds.<br>- `tree_method='hist'`: Uses histogram-based training for efficiency.<br>- `n_jobs=2`: Enables parallel processing.<br>- `verbosity=1`: Prints basic information during training.
**LightGBM**	- `class_weight='balanced'`: Adjusts weights for imbalanced classes.<br>- `max_depth=3`, `num_leaves=7`, `n_estimators=80`: Controls model complexity.<br>- `n_jobs=2`: Enables parallel training.<br>- `verbosity=1`: Provides training progress.<br>- `force_col_wise=True`: Optimises column-wise storage for efficiency.
**Support Vector Machine**	- `class_weight='balanced'`: Handles class imbalance.<br>- `probability=True`: Enables probability estimates for each class.
**Neural Network (NN)**	- *Input shape:* Number of input features.<br>- *3 layers:*<br>1st layer: `Dense(256, activation='relu')`, `BatchNormalization`, `Dropout(0.5)`<br>2nd layer: `Dense(128, activation='relu')`, `BatchNormalization`, `Dropout(0.5)`<br>3rd layer: `Dense(num_classes, activation='softmax')`<br>- `learning_rate=0.0005`: Adam optimizer learning rate.<br>- *Loss function:* `categorical_crossentropy`<br>- *Metrics:* `accuracy`<br>- `epochs=30`, `batch_size=32`<br>- `EarlyStopping`: patience=5, monitors `val_loss`.
**CNN**	- *Input shape:* Reshaped input for 1D CNN.<br>- *2 Conv1D layers:*<br>1st `Conv1D(64 filters, kernel_size=3, activation='relu')`<br>2nd `Conv1D(128 filters, kernel_size=3, activation='relu')`<br>- `MaxPooling`: `pool_size=2`<br>- `Flatten` layer before Dense layers<br>- Dense layers: `Dense(128, activation='relu')`, `Dropout(0.5)`<br>- `Output layer`: `Dense(num_classes, activation='softmax')`<br>- *Loss function:* `categorical_crossentropy`<br>- *Metrics:* `accuracy`<br>- `epochs=30`, `batch_size=32`<br>- `EarlyStopping`: patience=5, monitors `val_loss`.

12. D. A. Case, K. Belfon, T. A. Darden, R. E. Duke, G. Giambasu, T. J. Giese, M. K. Gilson, H. Gohlke, A. W. Goetz, R. Harris, S. Izadi, S. A. Izmailov, C. Jin, K. Kasavajhala, A. Kovalenko, T. S. Lee, S. LeGrand, P. Li, C. Lin, J. Liu, T. Luchko, R. Luo, D. J. Mermelstein, K. M. Merz, Y. Miao, G. Monard, C. Nguyen, H. Nguyen, I. Omelyan, A. Onufriev, F. Pan, S. Pantano, R. Qi, A. Rahnamoun, D. R. Roe, A. Roitberg, C. Sagui, S. Schott-Verdugo, J. Shen, C. L. Simmerling, N. R. Skrynnikov, J. Smith, J. Swails, R. C. Walker, J. Wang, H. Wei, R. M. Wolf, X. Wu, Y. Xue, D. M. York, S. Zhao, and P. A. Kollman. AMBER 2022. *University of California, San Francisco*, 2022.
13. J. A. Maier, C. Martinez, K. Kasavajhala, L. Wickstrom, K. E. Hauser, and C. Simmerling. ff14SB: Improving the accuracy of protein side chain and backbone parameters from ff99SB. *Journal of Chemical Theory and Computation*, 11(8):3696–3713, 2015.
14. W. L. Jorgensen, J. Chandrasekhar, J. D. Madura, R. W. Impey, and M. L. Klein. Comparison of simple potential functions for simulating liquid water. *The Journal of Chemical Physics*, 79(2):926–935, 1983.
15. H. J. C. Berendsen, J. P. M. Postma, W. F. van Gunsteren, A. Di Nola, and J. R. Haak. Molecular dynamics with coupling to an external bath. *The Journal of Chemical Physics*, 81(8):3684–3690, 1984
16. T. Darden, D. York, and L. Pedersen. Particle mesh Ewald: An N-log(N) method for Ewald sums in large systems. *The Journal of Chemical Physics*, 98(12):10089–10092, 1993.
17. S. Miyamoto and P. A. Kollman. SETTLE: An analytical version of the SHAKE and RATTLE algorithms for rigid water models. *Journal of Computational Chemistry*, 13(8):952–962, 1992.
18. V. Hornak, A. Okur, R. C. Rizzo, and C. Simmerling. HIV-1 protease flaps spontaneously open and reclose in molecular dynamics simulations. *Proceedings of the National Academy of Sciences of the United States of America*, 103(4):915–920, 2006.
19. G. A. Tribello, M. Bonomi, D. Branduardi, C. Camilloni, and G. Bussi. PLUMED 2: New feathers for an old bird. *Computer Physics Communications*, 185(2):604–613, 2014.

Table 7. Comparison of Different Performance Metrics of ML Models by Class for the GraphSage Embedding Method on 50,000 Frames for ADK. This frame size is suggested to users.

ML Model	Class	Precision	Recall	F1-Score	AUC	Class Accuracy
Logistic Regression	A	0.38	0.78	0.51	0.87	0.78
	B	0.89	0.98	0.94	1.00	0.98
	I	0.42	0.92	0.57	0.98	0.92
	N	0.93	0.67	0.78	0.88	0.67
	**Accuracy**			0.73		
Random Forest	A	0.49	0.45	0.47	0.89	0.45
	B	0.90	0.98	0.94	1.00	0.98
	I	0.57	0.72	0.63	0.99	0.72
	N	0.87	0.86	0.87	0.90	0.86
	**Accuracy**			0.82		
XGBoost	A	0.55	0.34	0.42	0.90	0.34
	B	0.93	0.98	0.95	1.00	0.98
	I	0.60	0.78	0.67	0.99	0.78
	N	0.86	0.90	0.88	0.91	0.90
	**Accuracy**			0.84		
CNN	A	0.49	0.45	0.47	0.89	0.45
	B	0.93	0.96	0.95	1.00	0.96
	I	0.59	0.73	0.65	0.99	0.73
	N	0.87	0.87	0.87	0.90	0.87
	**Accuracy**			0.82		
Neural Network	A	0.60	0.52	0.56	0.92	0.52
	B	0.93	0.97	0.95	1.00	0.97
	I	0.68	0.72	0.70	0.99	0.72
	N	0.89	0.91	0.90	0.93	0.91
	**Accuracy**			0.86		
LightGBM	A	0.53	0.38	0.44	0.90	0.38
	B	0.92	0.98	0.95	1.00	0.98
	I	0.57	0.79	0.66	0.99	0.79
	N	0.87	0.89	0.88	0.91	0.89
	**Accuracy**			0.83		
Support Vector	A	0.56	0.50	0.53	0.91	0.50
	B	0.92	0.98	0.95	1.00	0.98
	I	0.61	0.76	0.68	0.85	0.76
	N	0.89	0.89	0.89	0.90	0.89
	**Accuracy**			0.84		

Table 8. Comparison of class-specific and overall accuracy for trajectory sizes 7000, 17500, 35000, and 70000 using different machine learning models for GraphSAGE. Results are reported for the second system, *PlnE*.

Trajectory size	ML Model	Class A	Class B	Class N	Model Accuracy
7000	Logistic Regression	0.90	0.85	0.77	0.82
	Random Forest	0.89	0.81	0.86	0.86
	XGBoost	0.88	0.79	0.87	0.86
	LightGBM	0.88	0.80	0.86	0.85
	Neural Network	0.94	0.82	0.82	0.85
	CNN	0.86	0.81	0.85	0.85
	Support Vector	0.93	0.88	0.82	0.85
17500	Logistic Regression	0.91	0.89	0.77	0.82
	Random Forest	0.90	0.81	0.89	0.88
	XGBoost	0.90	0.83	0.89	0.88
	LightGBM	0.91	0.84	0.88	0.88
	Neural Network	0.95	0.87	0.81	0.85
	CNN	0.86	0.80	0.90	0.87
	Support Vector	0.93	0.91	0.83	0.87
35000	Logistic Regression	0.91	0.89	0.76	0.82
	Random Forest	0.90	0.85	0.89	0.89
	XGBoost	0.91	0.86	0.88	0.89
	LightGBM	0.91	0.87	0.87	0.88
	Neural Network	0.96	0.85	0.81	0.86
	CNN	0.90	0.83	0.90	0.89
	Support Vector	0.95	0.92	0.83	0.87
70000	Logistic Regression	0.91	0.89	0.76	0.82
	Random Forest	0.91	0.87	0.90	0.90
	XGBoost	0.92	0.88	0.89	0.90
	LightGBM	0.93	0.90	0.87	0.89
	Neural Network	0.93	0.89	0.82	0.86
	CNN	0.89	0.87	0.91	0.90
	Support Vector	0.95	0.93	0.84	0.88

Table 9. Comparison of Different Performance Metrics of ML Models by Class for the GraphSage Embedding Method on 70,000 Frames. Results are reported for the second system, *PlnE*.

ML Model	Class	Precision	Recall	F1-Score	AUC	Class Accuracy
Logistic Regression	A	0.79	0.91	0.85	0.98	0.91
	B	0.62	0.89	0.73	0.96	0.89
	N	0.92	0.76	0.83	0.93	0.76
	**Accuracy**			0.82		
Random Forest	A	0.88	0.91	0.90	0.99	0.91
	B	0.82	0.87	0.84	0.98	0.87
	N	0.93	0.90	0.91	0.97	0.90
	**Accuracy**			0.90		
XGBoost	A	0.88	0.92	0.90	0.99	0.92
	B	0.81	0.88	0.84	0.98	0.88
	N	0.93	0.89	0.91	0.96	0.89
	**Accuracy**			0.90		
Neural Network	A	0.82	0.93	0.87	0.99	0.93
	B	0.72	0.89	0.80	0.98	0.89
	N	0.94	0.82	0.88	0.95	0.82
	**Accuracy**			0.86		
CNN	A	0.90	0.89	0.89	0.99	0.89
	B	0.82	0.87	0.84	0.98	0.87
	N	0.92	0.91	0.91	0.96	0.91
	**Accuracy**			0.90		
Support Vector	A	0.84	0.95	0.89	0.99	0.95
	B	0.74	0.93	0.83	0.98	0.93
	N	0.96	0.84	0.89	0.96	0.84
	**Accuracy**			0.88		
LightGBM	A	0.86	0.93	0.89	0.99	0.93
	B	0.77	0.90	0.83	0.98	0.90
	N	0.94	0.87	0.90	0.96	0.87
	**Accuracy**			0.89		

Table 10. Comparison of class-specific and overall accuracy for different trajectory sizes using different machine learning models for GraphSAGE. Results are reported for the third system, *HIV-1 Protease*.

Trajectory size	ML Model	Class A	Class B	Class N	Model Accuracy
5000	Logistic Regression	0.36	0.79	0.92	0.89
	Random Forest	0.06	0.85	0.96	0.93
	XGBoost	0.27	0.89	0.95	0.93
	LightGBM	0.36	0.90	0.93	0.91
	Neural Network	0.42	0.89	0.92	0.90
	CNN	0.18	0.80	0.96	0.92
	Support Vector	0.21	0.86	0.96	0.93
12500	Logistic Regression	0.34	0.71	0.94	0.90
	Random Forest	0.13	0.85	0.96	0.93
	XGBoost	0.48	0.88	0.94	0.92
	LightGBM	0.66	0.93	0.90	0.90
	Neural Network	0.46	0.95	0.93	0.92
	CNN	0.39	0.94	0.93	0.92
	Support Vector	0.40	0.89	0.96	0.94
25000	Logistic Regression	0.39	0.79	0.93	0.90
	Random Forest	0.19	0.87	0.96	0.93
	XGBoost	0.42	0.89	0.95	0.93
	LightGBM	0.66	0.93	0.90	0.90
	Neural Network	0.68	0.97	0.90	0.91
	CNN	0.47	0.87	0.95	0.93
	Support Vector	0.51	0.90	0.95	0.93
50000	Logistic Regression	0.53	0.87	0.93	0.92
	Random Forest	0.26	0.90	0.95	0.93
	XGBoost	0.63	0.92	0.94	0.93
	LightGBM	0.82	0.94	0.89	0.89
	Neural Network	0.84	0.96	0.91	0.92
	CNN	0.39	0.87	0.97	0.94
	Support Vector	0.66	0.92	0.95	0.94

Table 11. Comparison of Different Performance Metrics of ML Models by Class for the GraphSAGE Embedding Method on 50000 Frames. Results are reported for the third system, *HIV-1 Protease*.

ML Model	Class	Precision	Recall	F1-Score	AUC	Class Accuracy
Logistic Regression	A	0.35	0.53	0.42	0.96	0.53
	B	0.76	0.87	0.81	0.98	0.87
	N	0.97	0.93	0.95	0.96	0.93
	**Accuracy**			0.92		
Random Forest	A	0.47	0.26	0.33	0.98	0.26
	B	0.78	0.90	0.83	0.99	0.90
	N	0.96	0.95	0.96	0.97	0.95
	**Accuracy**			0.93		
XGBoost	A	0.41	0.63	0.50	0.98	0.63
	B	0.77	0.92	0.84	0.99	0.92
	N	0.98	0.94	0.96	0.97	0.94
	**Accuracy**			0.93		
LightGBM	A	0.29	0.82	0.43	0.97	0.82
	B	0.70	0.94	0.80	0.98	0.94
	N	0.99	0.89	0.93	0.97	0.89
	**Accuracy**			0.89		
Neural Network	A	0.41	0.84	0.55	0.98	0.84
	B	0.72	0.96	0.83	0.99	0.96
	N	0.99	0.91	0.95	0.98	0.91
	**Accuracy**			0.92		
CNN	A	0.56	0.39	0.46	0.97	0.39
	B	0.84	0.87	0.86	0.99	0.87
	N	0.96	0.97	0.97	0.97	0.97
	**Accuracy**			0.94		
Support Vector	A	0.55	0.66	0.60	0.82	0.66
	B	0.81	0.92	0.86	0.99	0.92
	N	0.98	0.95	0.97	0.97	0.95
	**Accuracy**			0.94		

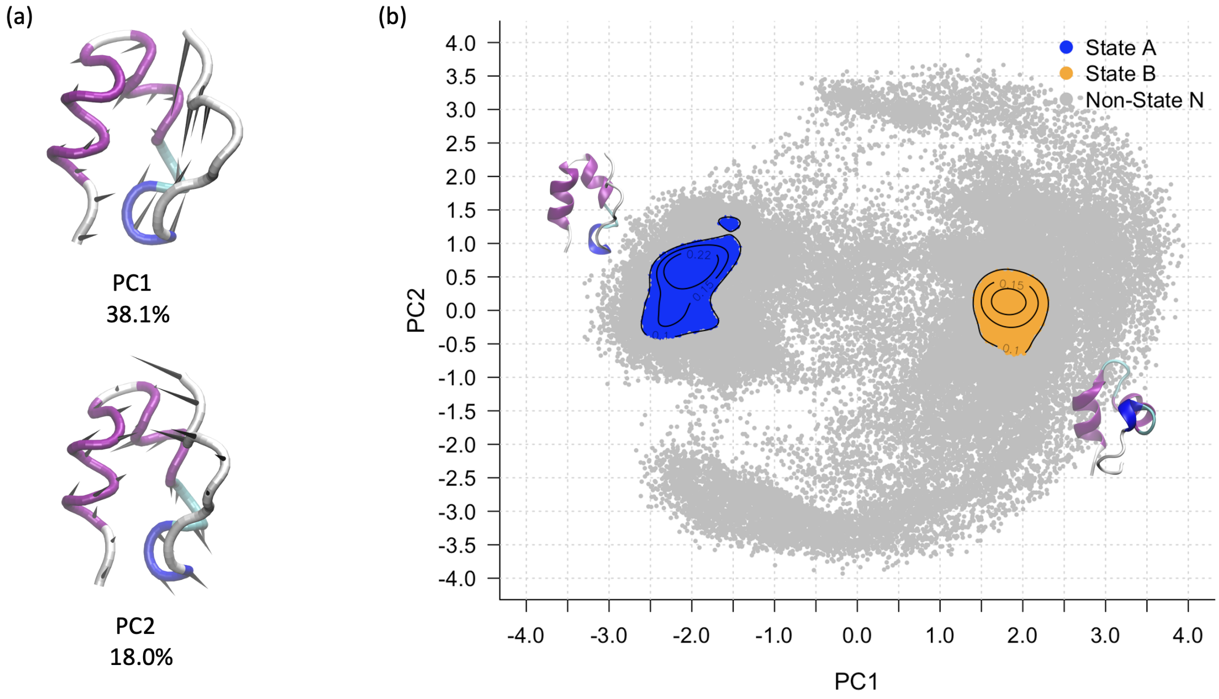

Fig. 6. The machine learning target for the second case study is derived from Principal Component Analysis (PCA) of the PlnE system. The first two principal components capture the main motions of the system, representing the dominant conformational transitions. These components are used to define state labels that guide the supervised learning process. The following colour scheme is used to represent the secondary structure of PlnE: **purple** indicates the α-helical regions spanning residues 13–21 and 25–32, while **white** denotes the coil or unstructured segments corresponding to residues 1–10 and 22–24. In addition, a short $\mathbf{3_{10}}$**-helix**, coloured **blue**, is present between residues 11 and 12, further contributing to the dynamic conformational profile of the peptide.

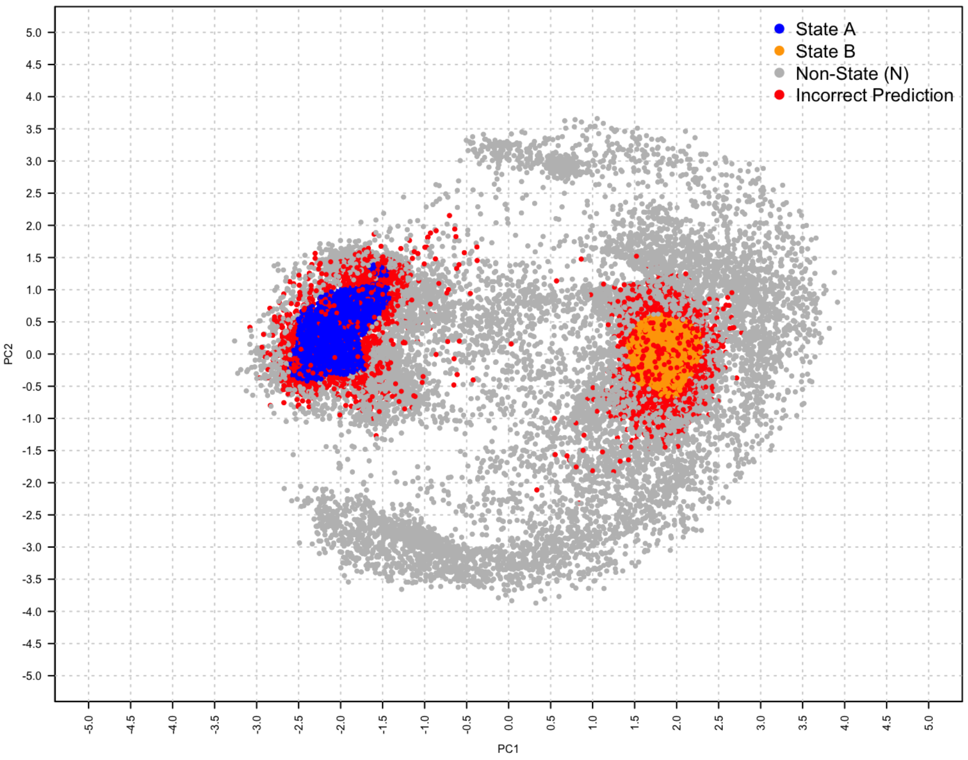

Fig. 7. The PCA plot shows the neural network's state predictions for 70000 frames, using color coding to distinguish between PlnE states. Red dots indicate incorrect predictions.

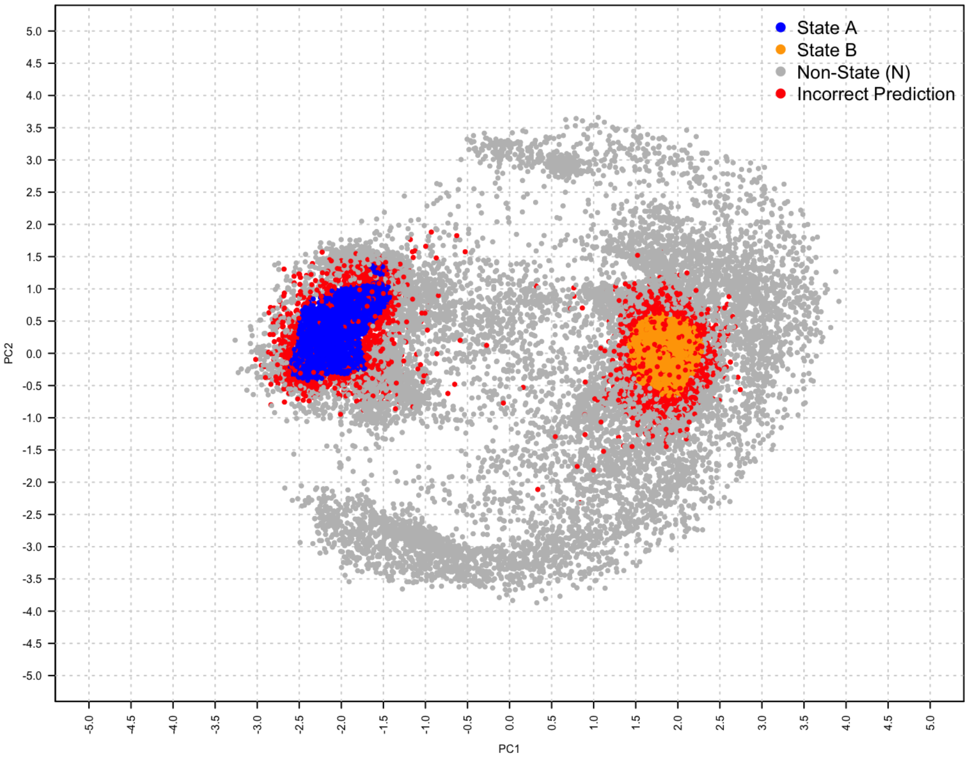

Fig. 8. The PCA plot shows the Support Vector's state predictions for 70000 frames, using color coding to distinguish between PlnE states. Red dots indicate incorrect predictions.

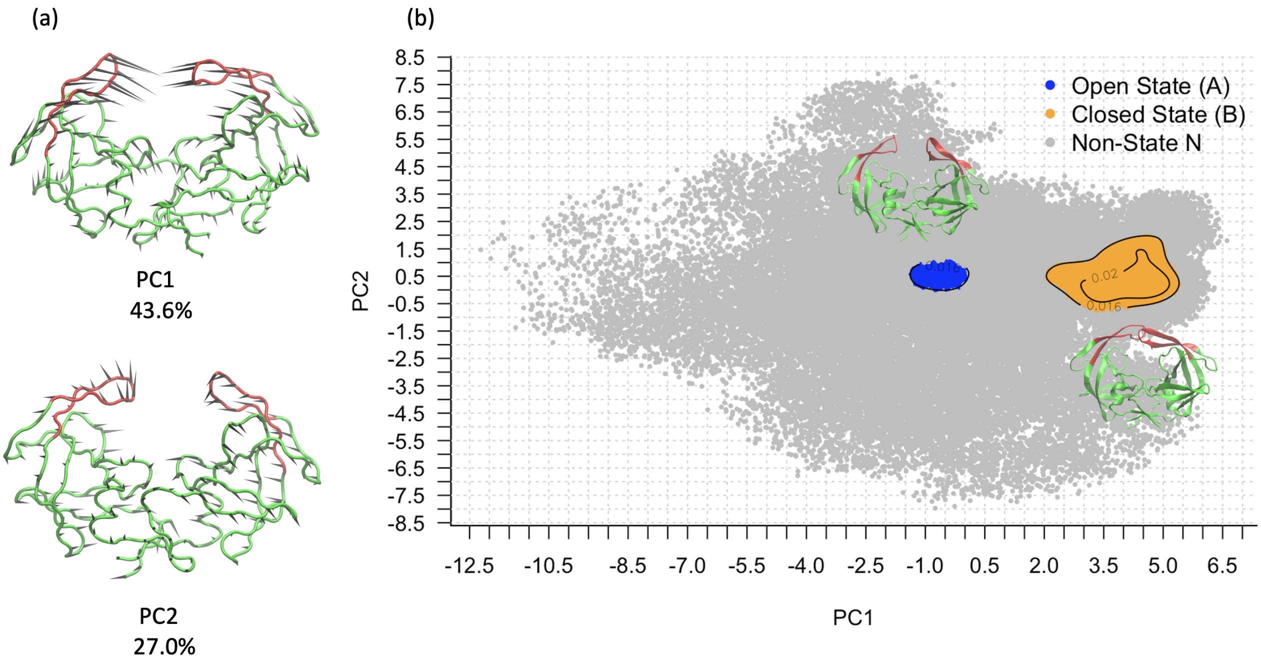

Fig. 9. The machine learning target for the third case study is derived from Principal Component Analysis (PCA) of the HIV-1 Protease system. Principal Component Analysis (PCA) of HIV-1 Protease dynamics reveals two dominant modes of motion. PC1 captures the characteristic loop opening and closing — a functionally critical movement that regulates substrate access to the active site. PC2 reflects a vertical breathing or hinge-bending motion, which may facilitate conformational transitions and enhance the flexibility of the binding pocket. In the structural representations, residues 44–55 and 143–154 correspond to the flexible loops and are coloured red to highlight their dynamic role in regulating active site accessibility. The remaining residues are shown in green, representing the more structurally stable regions of the HIV-1 Protease.

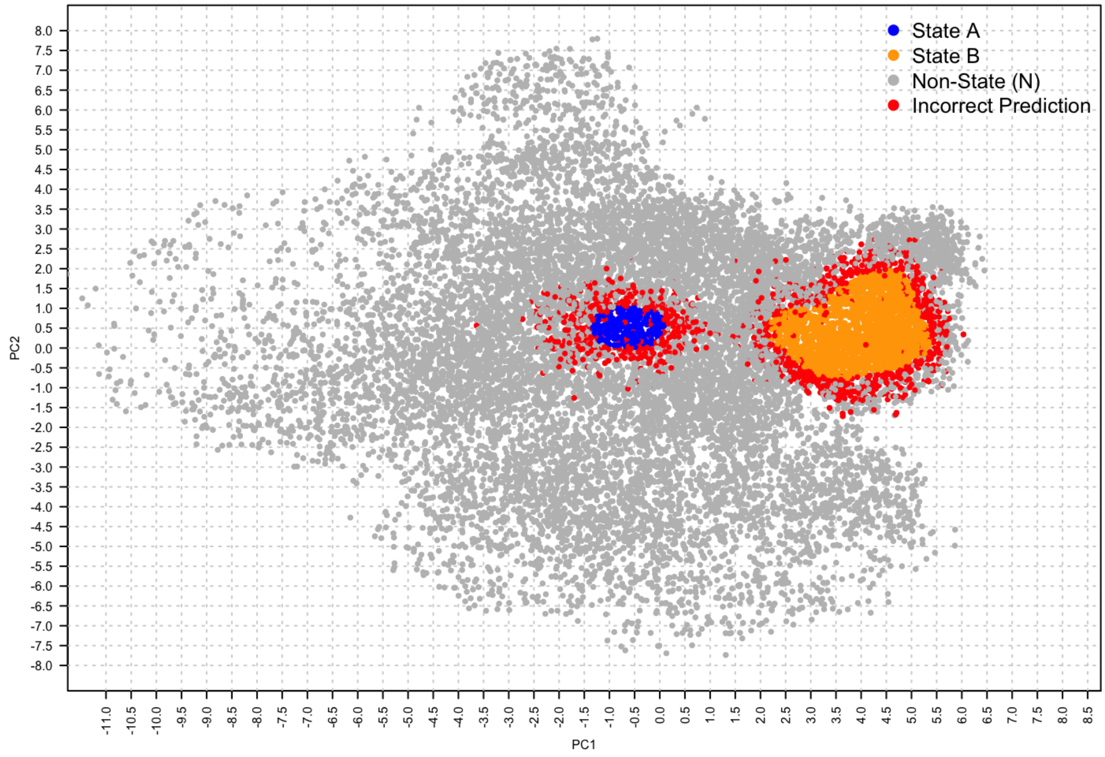

Fig. 10. The PCA plot shows the neural network's state predictions for 50000 frames, using color coding to distinguish between HIV-1 Protease states. Red dots indicate incorrect predictions.

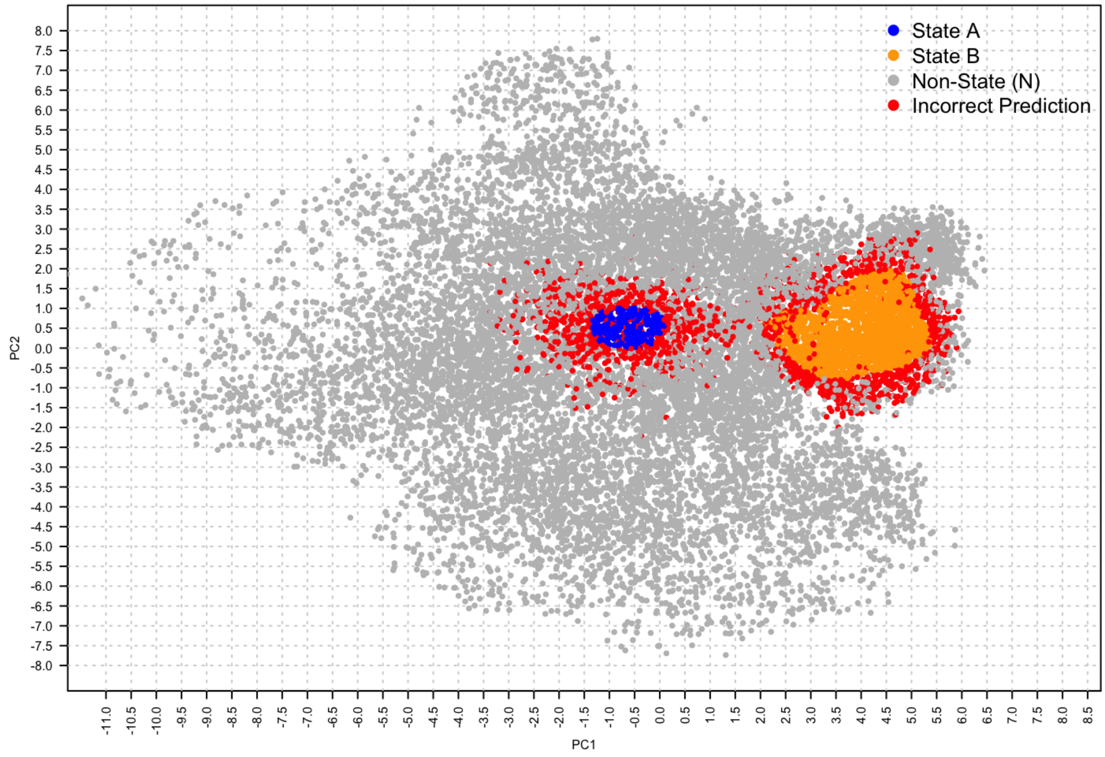

Fig. 11. The PCA plot shows the LightGBM's state predictions for 50000 frames, using color coding to distinguish between HIV-1 Protease states. Red dots indicate incorrect predictions.
